# Effects of Point Mutations in *Plasmodium falciparum* Dihydrofolate Reductase and Dihydropterate Synthase Genes on Clinical Outcomes and *In Vitro* Susceptibility to Sulfadoxine and Pyrimethamine

**DOI:** 10.1371/journal.pone.0006762

**Published:** 2009-08-26

**Authors:** David J. Bacon, Doug Tang, Carola Salas, Norma Roncal, Carmen Lucas, Lucia Gerena, Lorena Tapia, A. Alejandro Llanos-Cuentas, Coralith Garcia, Lelv Solari, Dennis Kyle, Alan J. Magill

**Affiliations:** 1 Parasitology Program, Naval Medical Research Center Detachment, Lima, Peru; 2 Department of Preventive Medicine and Biometrics, Uniformed Services University of the Health Sciences, Bethesda, Maryland, United States of America; 3 Experimental Therapeutics, Walter Reed Army Institute of Research, Silver Spring, Maryland, United States of America; 4 Universidad Peruana Cayetano Heredia, Lima, Peru; 5 Department of Global Health, College of Public Health, University of South Florida, Tampa, Florida, United States of America; London School of Hygiene & Tropical Medicine, United Kingdom

## Abstract

**Background:**

Sulfadoxine-pyrimethamine was a common first line drug therapy to treat uncomplicated falciparum malaria, but increasing therapeutic failures associated with the development of significant levels of resistance worldwide has prompted change to alternative treatment regimes in many national malaria control programs.

**Methodology and Finding:**

We conducted an *in vivo* therapeutic efficacy trial of sulfadoxine-pyrimethamine at two locations in the Peruvian Amazon enrolling 99 patients of which, 86 patients completed the protocol specified 28 day follow up. Our objective was to correlate the presence of polymorphisms in *P. falciparum dihydrofolate reductase* and *dihydropteroate synthase* to *in vitro* parasite susceptibility to sulfadoxine and pyrimethamine and to *in vivo* treatment outcomes. Inhibitory concentration 50 values of isolates increased with numbers of mutations (single [108N], sextuplet [BR/51I/108N/164L and 437G/581G]) and septuplet (BR/51I/108N/164L and 437G/540E/581G) with geometric means of 76 nM (35–166 nM), 582 nM (49-6890- nM) and 4909 (3575–6741 nM) nM for sulfadoxine and 33 nM (22–51 nM), 81 nM (19–345 nM), and 215 nM (176–262 nM) for pyrimethamine. A single mutation present in the isolate obtained at the time of enrollment from either *dihydrofolate reductase* (164L) or *dihydropteroate synthase* (540E) predicted treatment failure as well as any other single gene alone or in combination. Patients with the *dihydrofolate reductase* 164L mutation were 3.6 times as likely to be treatment failures [failures 85.4% (164L) vs 23.7% (I164); relative risk = 3.61; 95% CI: 2.14 – 6.64] while patients with the *dihydropteroate synthase* 540E were 2.6 times as likely to fail treatment (96.7% (540E) vs 37.5% (K540); relative risk = 2.58; 95% CI: 1.88 – 3.73). Patients with both *dihydrofolate reductase* 164L and *dihydropteroate synthase* 540E mutations were 4.1 times as likely to be treatment failures [96.7% vs 23.7%; RR = 4.08; 95% CI: 2.45 – 7.46] compared to patients having both wild forms (I164 and K540).

**Conclusions:**

In this part of the Amazon basin, it may be possible to predict treatment failure with sulfadoxine-pyrimethamine equally well by determination of either of the single mutations *dihydrofolate reductase* 164L or *dihydropteroate synthase* 540E.

**Trial Registration:**

ClinicalTrials.gov NCT00951106 NCT00951106

## Introduction

As the therapeutic efficacy of chloroquine for treatment of uncomplicated *P. falciparum* malaria declined in the late 1980s and early 1990s worldwide, sulfadoxine-pyrimethamine (SP; trade name Fansidar) was introduced as a replacement first line treatment. SP is an inexpensive fixed dose combination tablet which is well tolerated, highly efficacious, and can be administered in a single dose thus insuring compliance, making this drug ideal for first line therapy for uncomplicated malaria. However, drug resistance can occur rapidly [1], [2], [3] prompting ministries of health to change treatment policies [4]. SP was introduced as second line therapy in 1993 and first-line therapy in 1997 in the Amazon basin of Peru to counter widespread chloroquine resistance [5].

The mode of action of SP is well understood [6]. It is known that pyrimethamine and sulfadoxine preferentially bind to and inhibit the malaria parasite's dihydrofolate reductase (DHFR) and dihydropteroate synthase (DHPS) enzymes respectively, preventing *de novo* folic acid synthesis [7], [8]. The process of development of drug resistance against pyrimethamine occurs in a stepwise fashion commencing with the codon108N mutation in *Pfdhfr* followed by subsequent mutations at 50R, 51I, 59R and 164L [1]. A similar process occurs in *Pfdhps*, leading to resistance to sulfadoxine. Selection of antimalarial therapies that affect similar pathways within the host cell and malaria parasite is cause for concern but in the case of SDX and PYR, they appear to have little toxic effects on host cell survivability [9]. *In vivo* drug resistance in Peru was shown previously to be highly correlated with the presence of the DHFR haplotype 108N/51I/164L and DHPS haplotype 437G/540E/581G [10]. Insertion of the additional amino acids GKKNE at codon 30 of DHFR, termed the Bolivia repeat, has only been found in isolates from South America but a clear association of this repeat and drug sensitivity has not been shown [5], [11]. A single DHFR mutation at 59R along with a single DHPS mutation at 540E in certain parasite populations have been used to predict the presence of the quintuplet mutant and subsequent *in vivo* resistance [10] while in other population 437G is predictive [12], [13]. Interestingly, the polymorphism at 164L is uncommon in Africa [14], [15] which is contrary to South America were it is found at a high frequency in patients that fail SP treatment [5].

There are few published reports correlating patient outcomes following treatment with SP and *in vitro* drug susceptibility values obtained from *ex vivo* parasites [16]. This could be due to the difficulties associated with antimalarial therapies effecting *de no*vo folate synthesis since most cell culture medias contain folic acid and would necessitate the use of folate free media, which is costly [17].

Favorable treatment outcomes to antimalarials, including SP, are dependent on host immune responses and pharmacodynamics [18], [19], [20]. The combination of inadequate dosing and lack of acquired immunity among children especially, can give high treatment failure [21]. Therefore studies that evaluate *in vivo* and *in vitro* drug susceptibility of the same parasite isolates are needed to demonstrate key parasite specific factors that contribute to observed outcomes.

The purpose of the present study was to evaluate the therapeutic efficacy of SP in two locations in the Amazon rainforest region of Peru, and to correlate the presence of molecular markers associated with drug resistance and the Bolivia repeat sequence with *in vitro* drug susceptibility levels of sulphadoxine and pyrimethamine and treatment outcome. In vivo outcomes for one of these trials have been previously reported [22].

## Results

### Mutational analysis

We successfully genotyped all isolates collected during the enrollment process. The results of the genotyping produced three different two-locus haplotype for the DHFR/DHPS loci: single mutant (108N), sextuplet (BR/51I/108N/164L and 437G, 581G) or septuplet (BR/51I/108N/164L and 437G/540E/581G) with an overall frequency of 44%, 21% and 35%, respectively. No other two-locus haplotypes were identified. Genotypes of *Pf*DHFR and *Pf*DHPS and how they relate to clinical outcomes are summarized in Table 1. The presence of the sextuplet and septuplet were highly correlated with clinical failure (p<0.01). The Polymorphisms that were always present (108N) or always absent (A16, C50, C59, S436, and A613) provided no information on risk to treatment failure in this study (data not shown). Table 2 provides the treatment failure rates (and relative risk) depending on the number of mutations in DHFR, DHPS, and DHPF/DHFR. In all cases there is increasing risk of failure with numbers of mutations. The presence of 164L of DHFR and 540E of DHPS contained in the septuplet were an excellent predictor of treatment failures, with 96.7% of the patients having an isolates harboring this combination, failing treatment.

**Table 1:** Occurrence of mutations^1^ and treatment outcome [25].

Person No.		WHO 2003^1^	DHFR codon^2^				DHPS codon			No. of mutations (Total)^4^
	N		108N	51I	164L	BR^3^	581G	437G	540E	
1–29	29	ACPR	+							1
30–35	6	ACPR	+	+	+	+	+	+		6
36	1	ACPR	+							7
37–44	8	LPF	++							1
45–54	10	LPF	++	++	++	++	++	++		6
55–67	13	LPF	++	++	++	++	++	++	++	7
68	1	LCF	++	++	++	++	++	++	++	7
69	1	ETF	++							1
70–71	2	ETF	++	++	++	++	++	++		6
72–86	15	ETF	++	++	++	++	++	++	++	7
	86									

1 WHO 2003 definitions: ACPR, LPF, LCF, ETF.

2 Blank cells indicate wild –type sequence at the indicated codon; + indicate a mutation at the codon present in malaria isolates that were sensitive to SP; ++ indicate mutation present in malaria isolates that were resistant to SP.

3 Bolivian Repeat insert.

4 Genotypes: (1) DHFR 108/51/64 (with BR); (2) DHPS 581/437/540;

**Table 2:** Risk of treatment failure associated with occurrences of mutant alleles from DHPS, DHFR, and in combination.

	Treatment Outcome^1^					
*DHFR (all)*			Total	Percent failure^2^	RR^3^	95% CI^4^
No. of mutants	F	S				
108N	9	29	38	23.7	1	
BR/51I/108N/164L	41	7	48	85.4	3.61	2.14–6.64
Total	50	36	86	58.1	-	-
	Treatment Outcome^1^					
*DHPS (all)*			Total	Percent failure^2^	RR^3^	95% CI^4^
No. of mutants	F	S				
0	9	29	38	23.7	1	-
437G/581G	12	6	18	66.7	2.82	1.47–5.46
437G/540E/581G	29	1	30	96.7	4.08	2.45–7.46
Total	50	36	86	58.1	-	-
*DHPS/DHFR*	Treatment Outcome^1^					
(all)				Percent failure^2^	RR^3^	95% CI^4^
No. mutants	F	S	Total			
1	9	29	38	23.7	1	-
BR/51I/108N/164L and 437G/581G	12	6	18	66.7	2.82	1.47–5.46
BR/51I/108N/**164L** and 437G/**540E**/581G	29	1	30	96.7^5^	4.08	2.45–7.46
Total	50	36	86	58.1	-	-

1 “F”ailure = (LPF, LCF, ETF); “S”uccess = (ACPR).

2 Percent failure (no. failures/row total).

3 Relative risk of failure associated with mutant AA (relative to wild AA as reference) [RR = failure probability (mutant)/failure probability (wild)].

4 Confidence interval (for RR).

5 The presence of 164L of *Pf*DHFR and 540E *Pf*DHPS.

### *In vivo* efficacy outcomes

**Figure 1:**
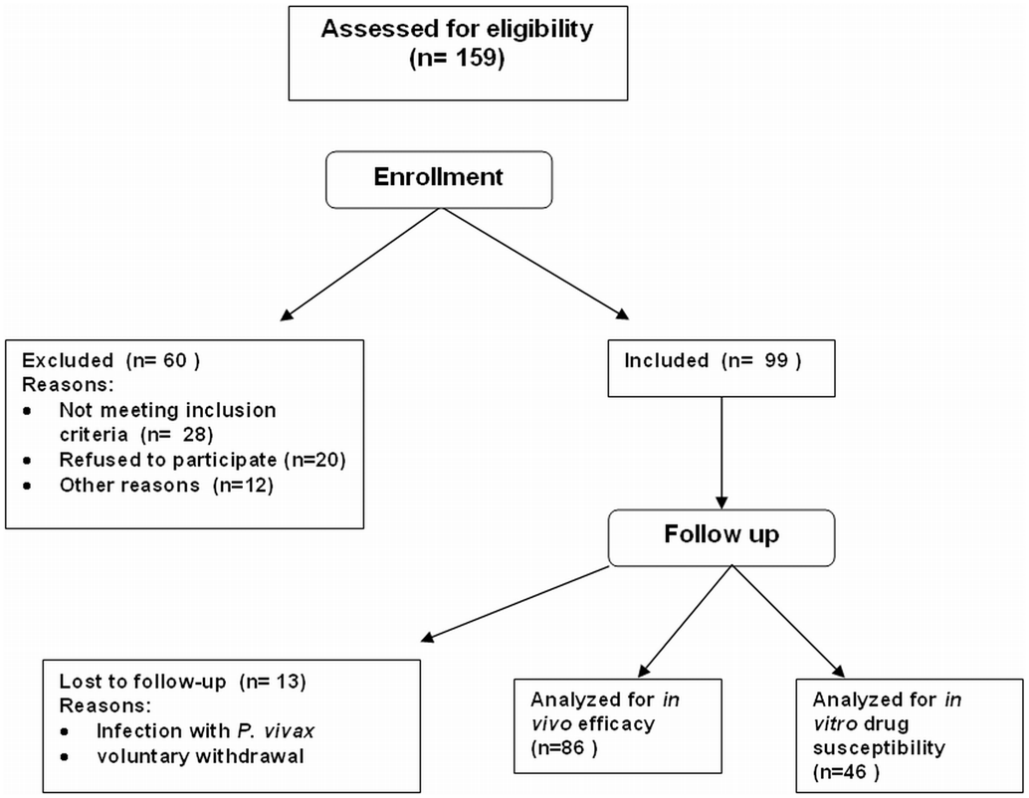
Flow chart of the enrollment process for the SP *in vivo* efficacy trial.

Of 159 patients assessed for eligibility, 99 were enrolled in the study (Figure 1) and 86 patients completed the study, of which, 52 were enrolled during the months of March through May 1999 in Padre Cocha and 34 were enrolled from Caballococha during July to August 1999 [22]. The original IRB approved protocol stated that clinical outcomes would be determined using the 1973 WHO system for classification [23] which focuses on parasitologic outcomes. During later analysis, the 2003 WHO system for categorizing outcomes, which includes a clinical component to the outcome category, was also assessed. Overall, the 2003 and 1973 WHO classification systems were in agreement, regardless of study site location (data not shown), in 99% (85/86) of the treatment outcomes [24]. Of the 36 persons who were treatment cures by at least one WHO criterion, 97% (35/36) were in agreement. There was no difference in the treatment cure rates (S% or ACPR %): 41% (35/86; 95% CI: 30.2%–51.8%) were successfully cured following treatment with SP using the 1973 method while 42% (36/86; 95% CI: 31.3%–52.9%) were cured when classified using the 2003 system [24]. There was also no difference in the times-to-treatment failure for the two WHO systems (data not shown). Because the two WHO classification systems gave comparable results, only the WHO 2003 classification results will be reported for subsequent analysis. Gametocyte densities were determined for all enrolled patients up to the time when they failed treatment. Patients that failed treatment with SP were no longer followed as part of this study, including weekly bleedings to determine gametocyte carriage rates. Therefore, 21 of the 22 patients from Padre Cocha classified as having ACPR and parasite clearance times (PCT) of 2–3 days following treatment, had gametocyte carriage densities determined by trained laboratory personnel on days 3, 7, 14 and 28. Nineteen of the 21 patients harbored a parasite with a single mutation in DHFR (108N) while two of 21 harbored parasites with the sextuplet haplotype (BR/51I/108N/164L and 437G/581G). In order to characterize transmission potential, the area under the curve (AUC) for the entire group classified as having ACPR (n = 21) was determined and calculated to be 3.8 [25]. Additionally, an average AUC for three separate groups, based on PCT, were determined. An AUC of 1.6 was calculated from one patient with a PCT = 1 day and 3.5 and 3.6 from patients with a PCT = 2 days (n = 10) and 3 days (n = 9), respectively. Eleven of the 19 patients (58%) harboring parasites containing the single mutation (108N) had gametocytes at day 28 (17–252 gametocytes/µl) while seven of the remaining eight had gametocyte present on day 21. One of the two parasites with the sextuplet haplotype had a 2-fold higher gametocyte density at day 14 compared to the rest of the group but this fell to 62 gametocytes/µl by day 28 while the remaining patient with the parasite harboring the sextuplet cleared gametocytes by day 7.

Isolates collected on the day of failure from all patients that failed treatment had 100% identical RFLP patterns for *Pfmsp2*, and DNA sequence identify for *Pfmsp1* block 2, when compared to isolates collected on day of enrollment, providing evidence that treatment failure was due to recrudescence not reinfection. At least nine genotypes of *Pfmsp2* as determined by PCR RFLP and seven genotypes of *Pfmsp1* block 2 as determined by DNA sequencing have been shown previously to be circulating in this region of Peru [26], [27]. We did not observe mixed infections from the DNA sequences, which has since been confirmed using a heteroduplex tracking assay [28]; Juliano and Meshnick, unpublished).

In addition, age, gender, weight, number of episodes of malaria in last year, species of previous malaria case and subsequent treatment of previous malaria case were not associated with treatment outcomes (data not shown). Additionally, serum was collected on the day of failure from 12 patients classified as ETF (PCT = 2–3 days) to determine drug levels of both SDX and PYR [29]. A median of 110±19 µg/ml (high of 157 µg/ml) for SDX and a median of 0.31±0.13 µg/ml (high of 0.6 µg/ml) for PYR were determined (data not shown).

### *In vitro* drug susceptibility results

**Figure 2:**
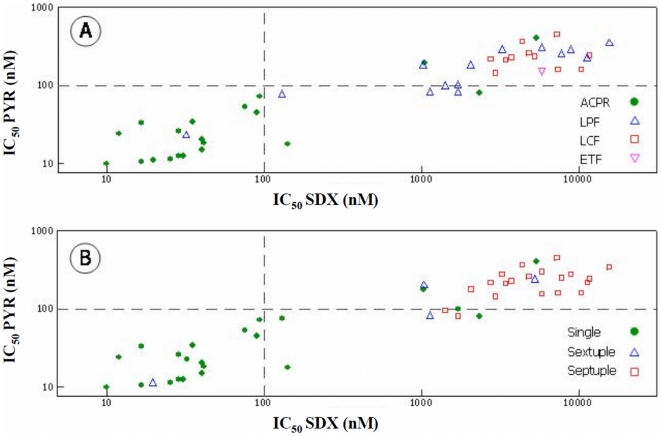
*In vitro* response (IC_50s_) of cultured adapted *P. falciparum* to sulfadoxine (SDX) and pyrimethamine (PYR) in relation to clinical outcomes (Panel A: ACPR •, LPF Δ, LCF □, ETF ▿) and number of mutation in DHFR and DHPS (Panel B: single •, sextuplet Δ, septuplet □).

Forty six isolates collected from Padre Cocha prior to treatment were adapted to *in vitro* culture and available for *in vitro* drug susceptibility testing. Twenty of the isolates were from patients classified as having ACPR (44%), 14 were from the LPF group (30%), 11 (24%) were from the ETF group and 1 was from the LCF group (2%). Figure 2 shows the IC_50_ values of SDX and PYR according to clinical outcome [24] (Figure 2A) and number of mutations (Figure 2B). In persons with ACPR, the geometric mean IC_50_ value for SDX was 62 nM (95% CI: 28–139 nM) and 29 nM (95% CI: 18–47 nM) for PYR. For persons who were treatment failures the geometric mean IC_50_ values were 1892 nM (LPF), 5701 nM (LCF) and 5104 nM (ETF) for SDX and 145 nM (LPF), 156 nM (LCF) and 231 nM (ETF) for PYR (Figure 2A).

**Figure 3:**
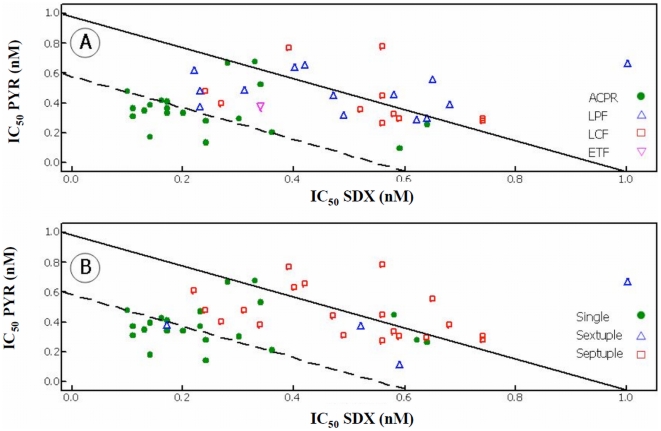
The derived isobolograms for the interaction of SDX and PYR and how they related to treatment outcome (Panel A: ACPR •, LPF Δ, LCF □, ETF ▿) and genotypes (Panel B: single •, sextuplet Δ, septuplet □). FIC<0.59 (dashed line) was classified as synergy; 0.6–1.5 was classified as additive (solid line) and >1.5 was classified as antagonism.

Therapeutic interactions between SDX and PYR were investigated by estimating FIC values from IC_50_ measurements taken in the presence of both drugs. When FIC values and clinical outcomes were compared (Figure 3A), all isolates classified as ACPR with IC_50_ values for SDX less than 85 nM (15/20; 80%) and one isolate from a patient classified as LPF had FIC values indicative of synergy (<0.6). The remaining parasites from the LPF, LCF, ETF groups and those classified as having ACPR with IC_50_ values greater than 85 nM had FIC values indicative of an additive effect between SDX and PYR (0.6–1.5). When FIC values were grouped based on the number of polymorphism (single, sextuplet and septuplet), the majority of the parasites with a single mutation showed synergy between the drug combination while nearly all those containing the sextuplet and septuplet haplotypes showed addition (Figure 3B).

## Discussion

Developing a cost effective malaria drug resistance surveillance program that can provide real time information and accurately predict the rate of treatment success is pivotal in the fight against this disease. Monitoring drug resistance worldwide is accomplished by *in vivo* drug efficacy trials, monitoring *in vitro* drug susceptibility values and detecting molecular markers. The easiest and most cost effective means of surveillance is monitoring the progression of polymorphisms associated with resistance, but the number of molecular markers currently known to confer resistance is limited. *In vivo* efficacy trials are the gold standard for determining drug resistance in real-time but these trials are costly and are not usually feasible for routine surveillance. *In vitro* drug susceptibility testing has been used extensively to determine sensitivity values but culturing of *ex vivo* parasites can be costly and difficult. We report a combination of all methods, including use of drug levels, and correlate the effects of polymorphism on *in vitro* IC_50_ values and *in vivo* efficacy.

In Peru, the presence of the septuplet haplotype (BR/51I/108N/164L and 437G/540E/581G) was a near absolute predictor of treatment failure. A limitation of the current study was the presence of only three two-locus haplotypes, making it impossible to discern the effects other haplotype combinations had on clinical outcomes in this region. In lieu of genotyping the entire DHFR and DHPS, we report that the presence of mutations at 164L of DHFR and 540E of *Pf*DHPS were excellent predictors of treatment failures. While the presence of the 164L mutation in Peruvian isolates is associated with high levels of SP resistance, it remains rare in Africa, despite being common in Asia and South America [15]. Interestingly, the presence of BR sequence was always associated with the 164L polymorphism. While the Bolivia repeat has been reported to be benign, the reason for such an insertion is unknown and currently has not been reported in Africa. On-going molecular studies using clinical isolates of *P. falciparum* collected in 2006 and 2007 from the Amazon basin region of Peru still show a strong association between the presence of the BR and the 164L polymorphism. However, unlike the study conducted in 1999, only 16% of the isolates have the BR and the 164L mutation which could be due to the loss of the selective pressure when SP was removed from the health clinics (Bacon et al, data not shown). Studies from Africa have shown that the triple mutant in DHFR (51I, 59R and 108N) is useful in predicting treatment failures but the widespread presence of 108N and the absence of 59R in Peru prevents the use of this haplotype combination for this means [30], [31], [32]. At the time this study was conducted, we did not identify isolates of *P. falciparum* with the 50R mutation but its presence has since been documented in other South American countries [5].

We found that not all patients harboring isolates with multiple mutations failed therapy, which could be due to the presence of some degree of acquired immunity as shown in a patient populations living in highly endemic regions [14], [33]. As an example, we documented that 7/36 (19%) patients that were successfully treated with SP, harbored isolates with six or greater mutations. Six of the seven subjects had reported at least one case of malaria in the past 12 months, with 4/6 reporting a case of *P. falciparum* and 2/6 reporting cases of *P. vivax* malaria. Two of the four reporting previous *P. falciparum* cases, reported receiving treatment with SP as first line therapy.

It has been shown that *P. falciparum* isolates collected in South America [5], [10], contain different haplotypes for the DHFR/DHPS loci as compared to isolates circulating in Africa [31], [34], [35] signifying a divergence of the 51I, 108N and 164L (South America) and 51I, 59R and 108N (African) haplotypes. Microsatellite analysis around the *Pfdhfr* and *Pfdhps* loci show that parasites from South America with mutations conferring mid- and high-level resistance to SP have a common origin [5] while African isolates are similar to those present in Southeast Asia [36]. Additional studies on the evaluation of the genetic variability around the highly resistant alleles in isolates from Africa provide evidence of a selective sweep attributed to selection of widespread use of pyrimethamine-sulfadoxine [37]. The presence of a limited number of haplotypes (single, sextuplet and septuplet) in Peru could be due to clonal expansion of a drug resistant parasite imported to the region from Brazil. With just a few haplotypes, selection of drug resistant phenotypes could have occurred, quickly resulting in a rapid progression of drug treatment failures and rapid removal of SP from the health centers. The ubiquitous mutation at 108N seen in this study is fixed as it is seen in all areas of Peru [38], regardless of treatment outcomes [10]. This mutation has been detected in several malaria endemic regions across Northern Peru, even in areas that still use SP as part of the national treatment policy for uncomplicated malaria [38]. While the 108N mutation appears to have no role in conferring resistance in Peru, it could permit for the persistence of gametocytes. In this study, 18 of the 19 patients with parasites harboring the single mutation and classified as ACPR, had gametocytes present 21–28 post treatment and mean AUC greater than 3.0. This finding possibly signifies that this mutation, while not singularly involved in conferring drug resistance, facilitates persistence that allows for a greater likelihood of transmission of genetically variant gametocytes to mosquitoes [13], [25]. Since resistance to SP occurs in a step-wise fashion with 108N being the first mutation in the series, transmission of gametocytes with this genotype could propagate further selection of a resistant genotype and phenotype [39]. This is an important issue, especially in regions where this combinational therapy is still being used. While we did not perform transmission studies, others have shown successful infectivity of mosquitoes with gametocytes with resistant genotypes that emerged following treatment with chloroquine [40] and SP [13].

Many published reports have described the effects of mutations in PfDHFR and PfDHPS on clinical success and failure following treatment with SP. However, very little is known if the effects of point mutations on *in vitro* drug susceptibility levels where the clinical outcomes are also known [16]. When using the threshold values published previously as IC_50_ values at or above 100 nM considered to be resistant [41], [42], we found that isolates with a single mutation at 108N in DHFR (22 isolates) had an IC_50_ geometric mean for PYR of 33 nM, which is lower than that reported previously [41]. Defining this subset of parasites with regards to the two-locus haplotype as having *in vitro* sensitive levels for PYR even though they have the 108N mutation, provides insight into the significance of this mutation and its usefulness at predicting *in vivo* efficacy in this region of the Amazon. The relationship between *in vitro* and *in vivo* resistance to SDX/PYR showed that resistance to SDX is directly related in most cases to treatment failure. This data is supported by the *in vitro* drug interaction studies where, as a group, the combination of SDX/PYR was synergistic in most of the parasites that were sensitive to therapy, whereas the drug combination was additive in all of the ETF/LPF/LCF outcomes (Figures 1 and 2). To fully understand drug resistance, determining the pharmacokinetics of various drug combination is essential [21]. With regards to SP, sulphadoxine is absorbed relatively slowly with a reported maximum concentration calculated to be up to 170 ug/ml (median 63.9 µg/ml) in a model median time of 24 h while pyrimethamine is absorbed quickly, reaching a reported maximum concentration of up to 1279 ng/ml (median 281 ng/ml) in a median time of 9.3 h [21]. The median values for both drugs are similar to those previously reported following administration of a standard dose of SP [29]. In this study, we report that 12 patients classified as ETF had median whole blood concentration from serum samples collected on day of failure (PCT = 2–3 days) for SDX and PYR of 110 µg/ml and 310 ng/ml, respectively. These values are similar to median values of 63.9 µg/ml and 281 ng/ml for SDX and PYR, respectively, previously published [21]. Given the half life of 6.7 days for SDX and PYR of 3.2 days [21], the serum levels obtained for this study were still within the therapeutic range even after 2–3 post treatment, which strongly suggests that failures were due to the presence of multiple mutation in DHFR and DHPS.

While complete publication of these results are occurring several years after the study was completed, the results of the *in vivo* efficacy trial were used by the Peruvian Ministry of Health, to support change in the national treatment policy for malaria [43]. What is relevant to on-going studies worldwide are the threshold of resistance for *in vitro* IC_50_ values and how they correlate with the various two-locus haplotypes. A bench mark can now be set to assist on-going surveillance for drug resistance against this combinational therapy in this region of the Amazon basin of South American. Continued surveillance in the absence of selective drug pressure, may reveal that SP may be suitable for future use in combination therapy in this region.

## Materials and Methods

### Study sites

A single *in vivo* therapeutic efficacy trial was conducted at two different sites located in low transmission regions of the Peruvian Amazon region in March-August 1999 when SP was recommended by the Peruvian National Malaria Control Program as first line treatment for uncomplicated malaria. One study site was located in the town of Padre Cocha, a village of 1400 inhabitants located on the Nanay River approximately five kilometers northwest of Iquitos, the capital of Loreto, Peru. A second study site was located in the town of Caballococha, which is approximately 300 km west of Padre Cocha, with a population of 3,300 and is located in the northeastern Peruvian Amazon region on the Peruvian border with Colombia and Brazil. The study was conducted following a protocol approved by the Walter Reed Army Institute of Research Human Use and Review Committee (Protocol No. 719) and by the Universidad Peruana Cayetano Heredia Ethics Committee report # CAR-017-DUIICT-99, under Protocol # 719. The protocol for this trial and supporting CONSORT checklist are available as supporting information; see checklist S1 and protocol S1.

### Patient enrollment

The procedure used for the *in vivo* drug efficacy study followed recommendations of the World Health Organization [24], [44]. Patients ≥6 month of age with suspected malaria were screened for malaria parasitemia with thick blood smears. There was no upper age limit designated for this study. Blood smears were stained with Giemsa and the parasite densities calculated by dividing the number of asexual parasites per 200 white blood cells and multiplying by the actual number of WBCs as determined by the QBC centrifugal hematology system (Becton Dickinson, Franklin Lakes, NJ). Patients presenting with *P. falciparum* asexual monoinfections, a parasite density greater than 500 parasites/µL but less than 200 parasites per oil immersion field (Ministry of Health, quantification of 4+), oral temperature ≥38.5°C (101°F) and/or a history of fever in the last 72 hours, and available and willing to return for follow-up were consented and enrolled in the trial using an IRB approved written consent form. In a subset of enrolled patients, gametocyte densities were determined on days 0, 2, 3, 4, 7, 14, 21 and 28 by counting sexual parasites per 200 WBCs. Patients that exhibited signs of severe malaria such as not being able to drink or breast feed, repeated vomiting, convulsions, lethargic or unconscious state, unable to sit or stand up. Respiratory distress or jaundice were not enrolled or followed clinically in this study but were referred to the local clinical care provides for hospitalization and treatment. Children under the age of 6 months were referred to the health clinic for diagnosis and treatment.

Sulfadoxine-pyrimethamine (Fansidar^®^; Roche S.A., Basel, Switzerland) was administered in a single oral dose equivalent to 1.25 mg/kg of pyrimethamine (up to a max of 75 mg) on Day 0 and were then followed with clinical histories, physical examinations, oral temperature measurements, and thick blood smears on days 1, 2, 3, 4, 7, 14, 21 and 28 to verify response. Patients that failed treatment with SP were treated on day of failure with a combination of quinine and tetracycline, quinine and chloroquine or quinine, tetracycline and primaquine.

### Treatment efficacy evaluation

Treatment outcomes were assessed using both the WHO standard definition for clinical [24] and parasitological responses [23]. To distinguish between recrudescence and reinfection, malaria parasites collected on day of enrollment (day 0) and day of recurrence were genotyped by RFLP analysis of a 700 bp PCR product of *Pfmsp2* [26] and DNA sequencing a 400 bp PCR product from *Pfmsp1* block 2 [27]. We previously reported that *Pfmsp1* block 2 is highly variable in this region of the Amazon basin, with at least eight different allelic variants circulating in the *P. falciparum* population, making it a suitable candidate for genotyping experimentation when used in conjunction with *Pfmsp2* [27].

### In vitro culturing of isolates

Fresh clinical *Plasmodium falciparum* isolates were cultured on average 8–12 weeks *in vitro* by a modification of the method of Trager and Jensen [45]. Parasitemia levels were determined by counting Giemsa-stain thin blood films. Parasites were initially adapted to culture in standard RPMI-1640. *P. falciparum* clones that are SP resistant (CDC/Indochina III (W-2), and sensitive CDC/Sierra Leone I (D6), [46] were used as reference standards.

### *In vitro* antimalarial drug susceptibility testing

The drug susceptibility test was based on the method described by Desjardins, *et al*., [47] with modifications developed by Milhous, et al. [48]. The antimalarials used in the study were obtained from the Division of Experimental Therapeutics, Walter Reed Army Institute of Research, Silver Spring, MD. Sulfadoxine and pyrimethamine were tested singly and in combination with six of the field isolates at fixed ratios of 1∶1, 1∶3, 3∶1, 1∶4, 4∶1, and 1∶5. The 50% inhibitory concentrations (IC_50's_) were determined for each drug alone and for fixed concentrations ratios by non-linear regression software–NFIT (University of Texas, Medical Branch, Galveston). IC_50_ values at or above 100 nM were considered indicative of resistance for both pyrimethamine and sulfadoxine [41], [42]. The IC_50_'s were used to calculate 50% fractional inhibitory concentrations (FIC_50_'s) singly and in combination as described by Berenbaum et al.[49]. The FIC_50's_ were expressed with the following equation FIC Drug A = IC_50A(B)_/IC_50A_, where IC_50A (B)_ is the 50% inhibitory concentration of drug A in the presence of drug B. The sum of FICs (∑FICs) of drug A and B at 1∶1 concentration ratio were used to determine the interaction of S/P in vitro. Isobolograms from a limited number of isolates defined the drug-drug interaction as follows: interactions were classified as synergism with sum FICs (ΣFICs) ≤0.6, as additive with ΣFICs 0.61–1.5 and antagonistic with ΣFICs >1.5 [50].

### Molecular markers determination to identify *Pfdhfr/Pfdhps* SNPs

Parasite DNA was extracted from 200 µL of whole blood collected on the day of enrollment from a three ml Vacutainer (Becton Dickson) with EDTA using QIAamp® DNA blood mini kit (Qiagen, Valencia, Ca) according to manufacturer's instructions. Five µl of *P. falciparum* genomic DNA was used for a PCR reaction using *Pfdhfr* and *Pfdhps* specific primers [32], [51]. PCR products were DNA sequenced to detect mutations A16V, S108T/N, C50R, N51I, C59R, I164L and the Bolivia repeat in *Pfdhfr* and S436A and A437G, K540E, A581G and A613T/S in *Pfdhps* [32], [51], [52].

### Statistical analysis

Statistical analyses were carried out using Minitab (Vs 14) and StatXact (Vs 4.01). Mean or geometric mean values were used to summarize quantitative measurements (age, weight, hemoglobin (Hb), drug IC_50_ values, parasite counts). Due to frequent travel for work, and the uncertainty where the cases of malaria were contracted, treatment outcomes from each site were combined. Unpaired t-tests (or one way ANOVA) were used to compare mean values in two (or > two) independent groups. Drug IC_50_ values and parasite counts were log transformed (base10) before analysis. Fisher's exact test was used to compare two independent proportions. McNemar's was be used to compare two dependent (paired) proportions. Multivariate logistic regression was used to help assess statistically significant predictors of treatment success/failure For single categorical predictor variables the relative risk (RR) for treatment failure and corresponding 95% confidence intervals (CIs) were computed. In all cases the reference group for assessing increased risk of failure is the category with the assumed lowest risk of treatment failure. For example, in assessing risk in relation to the total number of possible haplotypes in *Pfdhfr*/*dhps* (single, sextuplet and septuplet), the reference group is the category with a “single mutation”. Because of the close agreement between the two WHO systems for assessing therapeutic outcomes only the results using the clinical outcomes as defined in the WHO 2003 system are reported. All reported p-values are two-sided and 95% CIs were computed for important comparisons (effect measures).

## Supporting Information

Checklist S1 CONSORT Checklist
(0.03 MB DOC)

Protocol S1 Trial Protocol
(7.16 MB PDF)
