# Supplementary material for: Effects of Point Mutations in Plasmodium falciparum Dihydrofolate Reductase and Dihydropterate Synthase Genes on Clinical Outcomes and In Vitro Susceptibility to Sulfadoxine and Pyrimethamine
Source: PLoS One. 2009 Aug 26;4(8):e6762. doi: 10.1371/journal.pone.0006762 (PMC2728505; doi:10.1371/journal.pone.0006762)
Supplement: Checklist S1 — CONSORT Checklist (0.03 MB DOC) [file pone.0006762.s001.doc]

**The Consort E-Flowchart**

**Allocation**

**Analysis**

**Follow-Up**

**Enrollment**

Assessed for eligibility

( n= 225 )

Excluded ( n= 126 )

Too few or too many parasites ( n= 31 )

No available for follow up ( n=74)

Pregnant ( n= 4)

Not willing to give IC ( n= 4)

Received other study medications ( n=5)

Other ( n=8)

Allocated to intervention

( n= 99 )

Received allocated intervention

( n= 99 )

Did not receive allocated intervention

( n=0 )

Analyzed (n=86 )

Excluded from analysis (n= 0 )

Give reasons

Lost to follow-up ( n= 13 )

Emergent infection with *P. vivax* during the 28 day f/u period ( n=8)

Voluntary withdrawal or protocol violation ( n=5)

Is it Randomized?

**No**
