# Supplementary material for: Effects of Point Mutations in Plasmodium falciparum Dihydrofolate Reductase and Dihydropterate Synthase Genes on Clinical Outcomes and In Vitro Susceptibility to Sulfadoxine and Pyrimethamine
Source: PLoS One. 2009 Aug 26;4(8):e6762. doi: 10.1371/journal.pone.0006762 (PMC2728505; doi:10.1371/journal.pone.0006762)
Supplement: Protocol S1 — Trial Protocol (7.16 MB PDF) [file pone.0006762.s002.pdf]

Resistance to Chloroquine (CQ) and Pyrimethamine-Sulfadoxine (PS) for the Treatment of Uncomplicated *P. falciparum* Malaria in the Upper Amazon Basin.

WRAIR Protocol No. 719, HSPD Log No. A-8568: version 1.4 (18 Dec 98)

Principal Investigator(s):

Alan J. Magill M.D., LTC, MC, USA
Head, Parasitology
Naval Medical Research Center Detachment
Lima, Peru
51-1-561-1149 phone
51-1-561-3042 fax
magill@namrid.sld.pe

Alejandro Llanos Cuentas M.D
Head, School of Public Health
Universidad Peruana Cayetano Heredia
Lima, Peru
51-1-382-0310 phone
51-1-381-9072 fax
allanos@upch.edu.pe

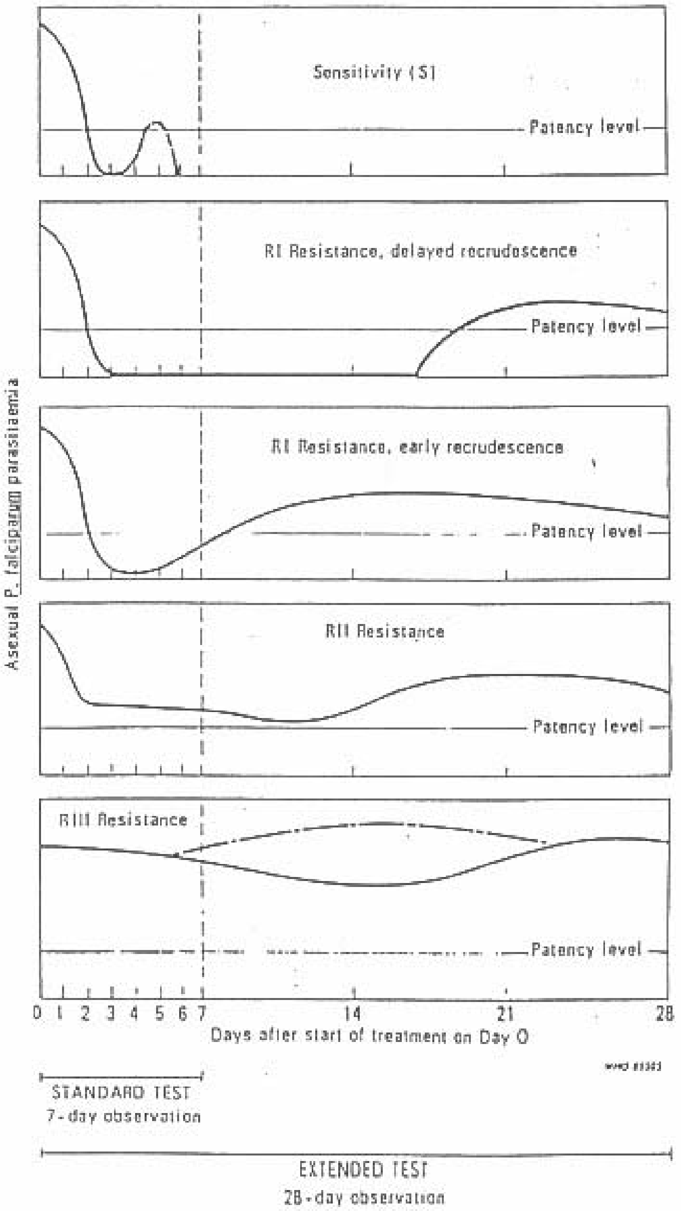

710 PROTOCOL–ENGLISH

PROJECT TITLE

Resistance to chloroquine (CQ) and pyrimethamine - sulfadoxine (PS) for the treatment of uncomplicated *P.* falciparum malaria in the Upper Amazon Basin

Short title: CQ/PS - resistance in the Peruvian Amazon

WRAIR Protocol N° 719 HSPD Log N° A-8568 Version 1.0 (18 Dec 98)

1. PROJECT TITLE: Resistance to chloroquine (CQ) and pyrimethamine-sulfadoxine (PS) for the treatment of uncomplicated *P. falciparum* malaria in the Upper Amazon Basin.

Short title: CQ/PS-resistance in the Peruvian Amazon

WRAIR Protocol No. 719, HSPD Log No. A-8568: version 1.0 (18 Dec 98)

2. PERSONNEL:

A) Principal Investigator:

Alan J. Magill M.D.
LTC, MC
Head, Parasitology
Naval Medical Research Detachment (NAMRID); Lima, Peru
011-51-1-561-1149 phone
011-51-1-561-3042 fax
magill@namrid.sld.pe

B) Associate Investigators:

Alejandro Llanos M.D.
Head, School of Public Health
Universidad Peruana Cayetano Heredia (UPCH)
Lima, Peru

Lely Solari M.D.
Clinical Research Associate
US NAMRID and Universidad Peruana Cayetano Heredia

LCDR Ellen Anderson
Parasitologist
US NAMRID

Hugo Rodriguez
Director del Programa de Control de Malaria y OEM
Hospital de Apoyo Yurimaguas
Yurimaguas, Peru

To be Named
Coordinator, Regional Malaria y OEM
Direccion Regional de Salud de Loreto
Iquitos, Peru

C) Collaborators:

MAJ Dennis Kyle
Chief, Dept. of Parasitology, Division of Experimental Therapeutics
Walter Reed Army Institute of Research (WRAIR)
Washington D.C. 20307

D) Approving Officials:

Carlos Calampa M.D.
Director de Regional de Salud Loreto
Iquiotos, Peru

Doug Watts Ph.D.
Scientific Director
U.S. NAMRID

Trueman Sharp M.D.
Officer-in-Charge
U.S. NAMRID

E) Medical Monitor:

Dr. Pedro Legua Leiva
Assisting Physician
Department of Communicable Diseases and Dermatology
Hospital Nacional Cayetano Heredia
Lima, Peru

3. RESOURCES AND COORDINATION: Physicians, technicians, and field workers from the US NAMRID, collaborating host country and US based institutions, and from the Ministry of Health (MINSA) of Peru will participate. US NAMRID and MINSA personnel, under the direction of the principal investigator (PI) will perform clinical therapeutic efficacy trials (*in vivo* resistance studies). MINSA personnel will assist in coordinating studies at all field sites. Blood samples collected during the therapeutic efficacy trials will be analyzed at NAMRID or in the laboratories of collaborating institutions.

4. LOCATION OF STUDY: The decision to test CQ or PS in any particular site will be made based on consultation with the local malaria control authorities from the Ministry of Health (MINSA). Current MINSA policy is to use chloroquine (CQ) as first line therapy for all areas in Peru with sensitive strains. Second line therapy is pyrimethamine / sulfadoxine (PS) in areas with CQ resistance. Third line therapy is quinine / tetracycline (QT) or quinine / clindamycin (QC) for areas with both CQ and PS resistance.

The decision to change form one therapy to another is based on follow-up of treated patients with control smears taken on days 7 and 14 following start of therapy. A smear positive slide on days 7 or 14 indicates failure and the patient is classified as a drug failure due to resistant parasites. This "cohorte" system is in use country-wide. When > 20% failures are documented in an area, this is cause for changing to the next line of therapy.

For example, if clinicians treating patients at the site report few if any treatment failures with CQ and a review of MINSA slide registries show a low number of parasitologic positives on the day 7 and day 14 control smears, one could conclude there is little evidence for CQ resistance in the area. This may be an ideal location to begin surveillance for emerging CQ resistance. On the other hand, a site with treatment failures and positive control slides may want a formal resistance evaluation to determine the real level of resistance in the community. These same considerations are true for PS. At this time we plan to perform three trials in the 1999 transmission season (Jan-Jul) in an area thought to be CQ and PS sensitive, an area thought to be CQ resistant and PS sensitive, and an area thought to be both CQ and PS resistant, but in which all parties want a formal assessment to determine the real scope of the problem. The decision to use any particular site must also consider the desire for the local MINSA to collaborate, the logistics of the site, and any relevant security considerations.

The choice between quinine (Q), quinine/tetracycline (QT), and quinine/clindamycin (QC) as an alternative treatment for those failing CQ or PS treatment will largely reflect the local standard of care in the community and the current MINSA guidelines. For most areas of Peru this means use of QT and QC for those in whom QT is contraindicated. Patients receiving alternative therapy will be monitored by study investigators in conjunction with MINSA health

care providers to insure a favorable outcome for the subject.

The endpoint for monitoring for the subject will be the conclusion of the 14 day or 28 day study period and the satisfactory clinical and parasitological response for all ETFs and LTFs who require alternative treatment. The endpoint for the location will be documentation of a greater than 20% failure rate. Continued surveillance may be performed with the next line of therapy. It is our plan to reevaluate selected drugs at the sites annually, in conjunction with the GEIS program, contingent on continued support.

NAMRID laboratories in Iquitos and/or Lima will receive blood samples from the various study sites and prepare, stain, and interpret blood smears; cryopreserve parasites; perform polymerase chain reaction (PCR); and perform *in vitro* culture and drug sensitivity assays. Isolates and filter paper blood dots will be shipped to participating laboratories in the United States and host country collaborating institutions.

5. INVESTIGATIONAL NEW DRUG (IND) INFORMATION: Not Applicable

6. TIME REQUIRED TO COMPLETE: Up to 4 years from the start of the study
Projected Start: January 1999; Projected Completion: January 2003.

7. INTRODUCTION:

A. Synopsis: *Plasmodium falciparum* malaria (Pf) has become a major problem in the Peruvian Amazon within the last 6 years. For example, only 729 cases of Pf were reported in the Department of Loreto in 1992, whereas 30,680 cases of Pf were reported during the first six months of 1997 (MINSA data on file). Significant numbers of clinical treatment failures to both CQ and pyrimethamine/sulfadoxine (PS) have been reported, although few rigorous studies have been performed. CQ is first line drug therapy for acute, uncomplicated Pf where it is still felt to be effective, and PS is second line therapy. PS is currently first line drug therapy for Pf in many areas of the Peruvian Amazon because of increasing clinical failures with CQ.

To assess the extent of CQ or PS resistance among Pf infected patients, prospective therapeutic efficacy trials will be performed at selected sites in the Peruvian Amazon. These studies will determine the clinical and parasitological response of Pf patients to standard therapy with CQ or PS. Symptomatic, febrile patients with smear confirmed Pf monoinfection will be enrolled. A short questionnaire will be administered to obtain pertinent demographic, clinical, and epidemiologic data upon enrollment. Blood will be obtained for Pf culture, drug levels, clinical lab parameters (e.g. CBC, Cr, ALT, etc.), serology, and PCR analysis. The patients will be evaluated on days 0 (pretreatment), 1, 2, 3, 4, 7, 14, 21 and 28 to verify response. When logistically feasible, patients will be followed for 28 days. When logistically difficult or impossible, the patient will be followed for 14 days. If a patient is found to be parasitemic at any time point between day 4 and the end of the study (day 14 or 28), additional blood will be drawn and the patient will then be treated with alternative antimalarial drugs (PS or quinine) as clinically indicated.

B. Military Relevancy: Malaria is the most important infectious disease threat to both US and Peruvian military forces in the Amazon. Failure of currently available antimalarial drugs to prevent clinical malaria (suppressive chemoprophylaxis) or to reliably cure soldiers once they become ill (therapy) are a major concern of the U.S. military infectious diseases research program (MIDRP). Research into the mechanisms of drug resistance, the correlation of *in vitro* markers to *in vivo* results, and the field evaluation of rapid methods for determining resistance are therefore relevant.

C. Objectives:

Primary:

1) Determine the level of CQ and / or PS resistance to *P. falciparum* in selected areas of the Upper Amazon by performing standard therapeutic efficacy studies.

Secondary:

1) Correlate the clinical response to standard therapy with mutations in the binding site for DHFR/DHPS inhibitors using mutation specific PCR.

2) Determine the DHFR/DHPS genotype of *P. falciparum* isolates in areas with little or no prior use of PS and areas with extensive use of PS.

3) Assess the relationship between specific sequences within the CQ resistance (CQ-R) locus of Pf chromosome 7 and *in vivo* resistance to CQ treatment.

4) Determine utility of selected non-microscopic malaria rapid diagnostic devices (MRDDs) to detect recurrent parasitemia in the follow-up of patients.

D. Status: The widespread use of CQ during the 1950s as part of the global malaria eradication program led to the near simultaneous appearance of chloroquine resistant *P. falciparum* (CRPF) malaria in Southeast Asia and South America in the early 1960s. CRPF has spread throughout much of the endemic areas of the world since then.(Payne 1987) PS was introduced in the early 1980s for the treatment of uncomplicated malaria in Southeast Asia and in the Amazon Basin. Resistance to pyrimethamine occurred quickly and resistant cases were documented in 1986 in Thailand. Drug resistance is defined as clinical failures, both therapeutic and prophylaxis, in a population taking a standard regimen. The basis of resistance in malaria is felt to be the selection (by drug pressure) of naturally occurring resistant parasites from among a population of "wild type" isolates as opposed to true acquired resistance of single isolates under continuous drug pressure. Assessment of CRPF is complicated by differences in the immune status of the host. For example, a failure of prophylaxis in a non-immune traveler, often the first sign of emerging resistance to a particular drug, is different from the failure of a drug regimen to cure a case of acute malaria in a semi-immune individual.

Traditionally, resistance has been defined by *in vivo* and *in vitro* methods. *In vivo* tests are also referred to as therapeutic efficacy trials. The first standardized methodology for the

assessment of *in vivo* response to drug therapy in Pf was developed shortly after the first reports of chloroquine resistance in this species.(1965; 1967) The test procedure has remained essentially unchanged since 1972.(1973) These therapeutic efficacy trials follow set criteria for the selection of patients, the administration of a standard treatment regimen of the appropriate drug, and examination of blood smears to determine a parasitological endpoint for the stipulated period, e.g. 7 days (standard WHO test) or 28 days (extended WHO test). The standardized tests were originally developed for CQ but they are also used for the evaluation of the response to other blood schizonticidal drugs (e.g. PS). While the value of these tests in the context of clinical trials is undisputed, their performance in the field met with constraints due to the need for daily blood examination, especially when follow-up needed to be extended beyond 7 days. In addition, these tests were primarily conceived for the assessment of the parasitological response of Pf in non-immune persons and took practically no note of the clinical response to the drugs.

This protocol incorporates the basic study plan of the current PAHO protocol "Assessment of Therapeutic Efficacy of Antimalarial Drugs for Uncomplicated Malaria in the Americas (OPS/HCP/HCT/113/98) which is based on that originally developed for the testing of the therapeutic efficacy of antimalarial drugs against clinically manifest infections with *Plasmodium falciparum* in infants and young children in areas of intense transmission (WHO document WHO/MAL/96.1077). In the development of this protocol, due note has been taken of earlier work towards the same objective, as reflected in WHO document WHO/MAL/94.1070, *Antimalarial Drug Policies.*

The original protocol and source of the current protocol is the WHO document WHO/MAL/96.1077. It was reviewed and endorsed in August 1996, at the intercountry workshop on "Malaria treatment and resistance in Kenya, Zambia and Malawi" (Mangochi, Malawi) when the inclusion of different follow-up periods, i.e. 7 days, 14 days or 28 days was recommended, with indication of their role and applications. This protocol was presented and reviewed at the "Interregional meeting on malaria control with emphasis on drug resistance", Manila, Philippines, October 1996, during which several modifications were suggested for its adaptation to areas with low or moderate transmission. The WHO protocol "Assessment of

Therapeutic Efficacy of Antimalarial Drugs for Uncomplicated Falciparum Malaria", draft 28.2.97 is based on these recommendations and represents a standard method for monitoring the therapeutic efficacy of antimalarial drugs for the treatment of patients suffering from uncomplicated malaria in areas of low or moderate transmission. Finally, PAHO protocol OPS/HCP/HCT/113/98 incorporates revisions and adaptations to conditions in the Americas from a meeting of regional experts held in Manaus, Brazil, in March, 1998.

The PAHO protocol OPS/HCP/HCT/113/98 takes into account clinical and parasitological response, the need for an efficient technique that provides accurate, reliable and representative results which lend themselves to inter-area comparison, and applicability under different epidemiological conditions. In the described form the test is simple and feasible, provides the essential information, requiring only modest resources in terms of staff and material.

The purpose of a therapeutic efficacy protocol is to determine the practical efficacy of a particular drug regimen, with the ultimate objective of ascertaining its continued usefulness or the need for replacing it in the routine treatment of uncomplicated falciparum malaria in a particular area. Performance in sentinel sites on an annual basis is an effective surveillance system.

Traditionally, *in vitro* methods of assessing resistance have been either the schizont maturation test (WHO microtest) adapted from the method of Rieckman(Rieckmann, Campbell et al. 1978) or a metabolic method utilizing the uptake of tritiated thymidine that is the basis for current drug sensitivity assays.(Desjardins, Canfield et al. 1979) Both methods have contributed greatly to the *in vitro* determination of resistance but each have significant limitations. The WHO microtest is labor intensive and performs poorly with low parasitemias or with prior antimalarial drug use. Drug sensitivity assays are technically and logistically difficult requiring Pf isolates adapted through *in vitro* culture and the use of radioisotopes. In addition, some loss of genetic diversity occurs during culture adaptation. Drug sensitivity assays are most useful for determining the sensitivity of type isolates to a variety of available and investigational drugs. Use of mutation specific PCR to identify drug resistant genotypes is a promising methodology to rapidly screen a relatively large number of isolates.

Advances in our understanding of the molecular basis of *in vitro* Pf resistance to CQ are leading to the definition of the molecular determinants of CQ-R.(Wellems, Walker-Jonah et al. 1991) Chloroquine resistance in P. falciparum cross maps as a Mendelian trait to a 36 kb segment of chromosome 7. This segment harbors cg2, a gene encoding a unique approximately 330 kDa protein with complex polymorphisms. A specific set of polymorphisms in 20 chloroquine resistant parasites from Asia and Africa, in contrast with numerous differences in 21 sensitive parasites, suggests selection of a cg2 allele originating in Indochina over 40 years ago. One chloroquine sensitive clone exhibited this allele, suggesting another resistance component. South American parasites have cg2 polymorphisms consistent with a separate origin of resistance. CG2 protein is found at the parasite periphery, a site of chloroquine transport, and in association with hemozoin of the digestive vacuole, where chloroquine inhibits heme polymerization.(Su, Kirkman et al. 1997)

The successful development of molecular assays for chloroquine resistance will aid in designing optimal antimalarial chemotherapy policies in the face of changing patterns of resistance. While studies in the Malaria Genetics section of the Laboratory of Parasitic Diseases at NIH have defined a molecular basis for *in vitro* resistance of *P. falciparum* to CQ, the relationship between putative CQ-R genotypes and *in vivo* CQ resistance can only be accomplished in clinical field studies. A definitive answer will require prospective studies with clear documentation of correct CQ use, lack of other antimalarial use, and contemporaneously collected samples representing CQ-sensitive (CQ-S) and CQ-R infections as well as collection of samples for correlation with results of *in vitro* drug sensitivity assays. Comparison of the CQ-R genotype of pretreatment infections with the clinical outcome of those infections will provide the basis for testing the hypothesis that the putative CQ-R genotypes are determinants of *in vivo* CQ resistance. Because it is possible that multiple genetic mechanisms may contribute to *in vivo* chloroquine resistance, samples obtained in this study will also be preserved for future studies evaluating alternative or additional CQ-R candidate genes. The extent of chloroquine resistance in most areas of Peru is unknown and the presence of potentially novel CQ-R genotypes may be present which will facilitate further studies of the genetic basis of CQ resistance.

Pf resistance to PS is associated with point mutations in the active site of the parasite enzymes dihydrofolate reductase (DHFR) and dihydropterate synthase (DHPS). Pyrimethamine binds and inhibits DHFR and sulfa drugs act on DHPS. The combination of a single point mutation in DHFR causing a serine to asparagine change at position 108 has been linked , either alone or in combination with mutations at positions 51, 59, or 164, to pyrimethamine resistance.(Cowman, Morry et al. 1988; Peterson, Walliker et al. 1988; Zolg, Plitt et al. 1989; Foote, Galatis et al. 1990; Peterson, Di Santi et al. 1991; Foote and Cowman 1994) Two new mutations in DHFR, an in-frame 15 bp repeat, termed the Bolivia repeat (BR), and a single base alteration at DHFR codon 50 have been found from isolates in the Amazon Basin of South America (C. Plowe - personal communication). Point mutations in DHPS that have been associated with decreased susceptibility to sulfadoxine include those at positions 436, 437, 581, and 613.

The relationship between the DHFR/DHPS mutations and *in vivo* resistance to PS remains incompletely characterized. Because PCR offers many advantages over standard *in vivo* resistance and *in vitro* drug sensitivity assays, molecular assays offer a potentially useful tool for surveillance of resistance. These assays are in need of validation as predictors of *in vivo* resistance. Mutation specific PCR methods have been developed to detect the presence of these genotypes in parasite DNA extracted from filter paper blood samples, using methods that have been shown to be feasible in malaria-endemic areas.(Plowe, Djimde et al. 1995; Plowe and Wellems 1995; Plowe, Djimde et al. 1996)

PS is currently the first line drug therapy for acute, uncomplicated *P. falciparum* malaria in many areas of the Peruvian Amazon because of increasing clinical failures with CQ. PS is the second line therapy in other areas where CQ is still felt to be effective. PS is an important component of the control program in Peru as it is inexpensive and can be given as a single treatment dose. Resistance to PS is not well documented in most areas of Peru, but widespread reports of clinical failures from health posts and clinicians indicate RII/RIII resistance is likely to be present in many areas of the Department of Loreto. Preliminary studies performed by personnel of US NAMRID and the MINSA have found 27% RII/RIII failures with PS in one location near Iquitos.(Leinicke, Lucas et al. 1997) Additionally, it has been

shown that a specific DHFR and DHPS genotype is strongly associated with *in vivo* PS resistance in the Pf epidemic in Peru (J Kublin - personal communication). We plan to determine the number and type of DHFR / DHPS mutations present in an area where little or no use of PS has yet occurred and monitor the rapidity of changes that occur when PS is introduced.

Factors such as immunity and drug bioavailability may impact on the therapeutic efficacy of CQ and PS independent of parasite resistance. Therefore, in the logistic regression analysis for risk factors for CQ-R and PS resistance, age, number of previous episodes of malaria, and area of residence will be analyzed as potential confounders contributing to the *in vivo* response to CQ or PS treatment. Age is chosen as an available marker for clinical immune status in this epidemiologic setting and area of residence is included to account for the possibility that levels of exposure are different among people residing in different areas, e.g. urban vs. rural. Sera will be collected and stored for potential analysis of more specific immune markers such as antibody responses to malarial antigens as surrogate markers for acquired immunity. Blood collected on filter paper from critical days will be used to determine drug levels to assess bioavailability.

Determination of the therapeutic efficacy of antimalarials and monitoring the efficacy of antimalarial drugs over time, especially in the most vulnerable population groups such as infants, young children, pregnant women, and nonimmune adults in areas with high endemicity of malaria, can help guide national antimalarial drug policy. In order to assess the therapeutic efficacy of routine treatment regimens the test will be carried out only in persons suffering from clinically manifest, microscopically confirmed Pf malaria. The clinical response of the patients is the main criterion and the number of parasitological examinations will be restricted to the minimum required for ensuring the patient's safety.

If a limited set of mutations can be identified as predictive of *in vivo* CQ or PS resistance, it will be possible to conduct broad surveillance for drug resistant strains of malaria. The molecular epidemiology studies resulting from such surveillance will be especially useful in areas where both CQ and PS are still in use, such as the Upper Amazon.

8. STUDY PLAN: Therapeutic efficacy trials performed with this protocol will be based on a protocol from the current World Health Organization (WHO) and Pan American Health Organization (PAHO) recommendations (Assessment of Therapeutic Efficacy of Antimalarial Drugs for uncomplicated falciparum malaria in the Americas, OPS/HCP/HCT/113/98). It is important that our studies are compatible with this protocol so that we may participate in programs throughout the region consistent with our mission in the DoD Global Emerging Infections Surveillance (GEIS) program. Studies consist of recording essential patient information, a clinical assessment, documenting parasitemia, supervised treatment with CQ or PS, clinical assessment on Days 0, 1, 2, 3, 4, 7, 14, 21 and 28, and parasitological examination on at least days 0, 1, 2, 3, 7, 14, and 28. (see Time and Event schedule, and flow sheet, Attachment No. 1).

On presentation to a MINSA medical treatment facility:

1) The patient (and prospective volunteer) will have a fingerstick performed by the local MINSA personnel to determine if the patient has a mono-infection with Pf malaria. The smear will be stained and interpreted on site by a study microscopist. If the patient is smear positive for Pf malaria he will be interviewed by a study team member in conjuction with local health care providers of the MINSA. If the patient meets the inclusion and exclusion criteria (see Inclusion / Exclusion Criteria, attachment No. 2) and agrees to sign a written informed consent (see Informed Consent, attachment No. 3), he will be offered enrollment into the study and assigned a study number. Individuals who are not positive by smear or who have Pv infection will return to the MINSA health care provider for further care as needed.

2) Day 0 (D0) data will be recorded in a case report form (CRF) (see CRF, attachment 4).

3) Venipuncture will be performed using standard aseptic technique suitable for "blood cultures" (See SOP for Venipuncture, attachment No. 5). An experienced phlebotamist will take the blood sample from an an antecubital vein . Approximately 12 ml (2.5 teaspoons) of whole venous blood will be obtained from each volunteer. The blood will be collected into 3

Vacutainer® collection tubes using the Vacutainer® system or a needle and syringe. One 5 ml tube with acid-citrate-dextrose (ACD) anitcoagulant ("yellow top") will be used to cryopreserve the Pf isolate, one 5 ml tube with no anticoagulant ("red top") will be used to collect serum for determination of antibody titers to malarial antigens and clinical parameters such as liver enzymes, and one 2.5 ml tube with ethylene diaminoacetic acid (EDTA) anticoagulant to determine a complete blood count.

4) Venous blood will also be used to prepare, stain, and interpret a blood film. In addition, non-microscopic assays will be performed with malaria rapid diagnostic devices (MRDDs). At least four products are likely to be considered Paraight™ F (Becton Dickinson Microbiology Systems, Sparks, MD, USA), Parasight F+V (Becton Dickinson Microbiology Systems, Sparks, MD, USA), Pf. combo (AMRAD-ICT, Sydney, Australia) and Optimal® (Flow, Inc., Porland, OR, USA). See Performance of Optimal, attachment No. 6 and Performance of Parasight F, attachment No. 7. Results of the respective MRDD are recorded on Attachments No. 8 and 9, respectively. The purpose of the dipsticks is to determine if they can be successfully used in the follow-up evaluations for parasitemia. Parasight F™, Parasight F+V, and Pf combo use Pf histidine rich protein (HRP-II) as the target antigen. Optimal® uses pLDH as the target antigen. We and others have found HRPII based tests remain positive for several days after the smear is negative (antigen positive, parasite negative). This attribute would not make these tests useful for the goal of detecting recrudescing parasites. On the other hand, pLDH is thought to correlate much more closely with parasitemia so these MRDDs may be useful in the detection of recrudescing parasites. At each encounter, fingerprick blood will be used to perform the MRDD assays on site, in real time. The investigator will record the results on the appropriate DRF and these results will be compared to the final micro readings at a later time. MRDD results will not be used to guide or determine treatment. Parasight F™ is available and licensed for use in Peru. To my knowledge, the other MRDDs are not. The PI has access to these MRDDs through other collaborative trials.

5) Patients will then be treated with a standard course of 25 mg (base) / kg of CQ over 3 days, 10 mg / kg on day 1 (D1), 10 mg / kg on D2, and 5 mg / kg on D3. All treatment doses will be given under supervision of a study investigator (directly observed therapy or DOT), and the

patient will be observed for at least 30 minutes post administration to ascertain retention of the drug. If the patient vomits within the first 30 minutes post administration, the treatment should be repeated with the same dose. Patients with persistent vomiting will be excluded from the study and referred to the appropriate health facility. Verification of CQ treatment will be documented on attachment No. 10. Verification of PS treatment will be documented on atttachment No. 11.

6) Subjects will return to the clinic on days 1, 2, 3, 4, 7, 14, 21 and 28. Subjects will have a fingerstick and malaria smear performed on any day in which "danger signs" are present (see inclusion / exlcusion criteria), days 3, 7, 14, and 28 or any other day in which the study physician feels a smear is clinically indicated.

7) Subjects will have a clinical evaluation performed on each encounter with the subject. Results are recorded on attachment No. 12.

8) Subjects or the parent/guardian will be instructed to bring the child to the clinic on any of the days 1-28 if he/she develops any of the danger signs, if the volunteer is still sick or if there is any cause for worry. At any time, if the volunteer shows clear clinical deterioration a blood film will be taken, in order to differentiate resistant malaria from other causes of treatment failure (see Overall Classification of Therapeutic Response, below).

9) Individuals who worsen clinically and require treatment with a different drug will be removed from the study after a second sample of venous blood is taken. Individuals who still have parasites in their blood smear on day 7 will be treated with a second line therapy {PS or quinine plus tetracycline (TCN)} regardless of the clinical status.

Classification of Therapeutic Response:

Parasitologic:

Parasitologic criteria for recognition of suspected resistance of malaria parasites to

antimalarial drug therapy were adopted in 1974. A standard procedure for determining the level of resistance was subsequently recommended by WHO. These were defined as:

RI resistance: disappearance of parasitemia by thick smear microscopy by day 7 after drug therapy followed by recurrent parasitemia by day 28;

RII resistance: a ≥75% diminution of parasitemia by day 3 and followed by a subsequent rise in parasitemia; and,

RIII resistance: a <75% or no diminution of parasitemia by day 2 (48 hours after first treatment dose) following drug therapy.

Therapeutic failure is defined as RII or RIII resistance and persistence or worsening of fever and other symptoms of malaria at day 3. (Note: day of treatment is day 0.)

Clinical:

There are three categories of therapeutic response recommended in the current PAHO protocol "Assessment of Therapeutic Efficacy of Antimalarial Drugs for Uncomplicated Malaria in the Americas (OPS/HCP/HCT/113/98): early treatment failure (ETF), late treatment failure (LTF) and adequate clinical response (ACR). These definitions were established in recognition of the difficulties encountered in using the traditional criteria. They are defined as follows:

Early treatment failure (ETF): if the patient develops one of the following conditions during the first three days of follow-up:

1. Development of "danger signs" or severe malaria on Day 1, Day 2, or Day 3 in the presence of parasitemia;

2. Parasitemia on Day 2 higher than Day 0 count;

3. Parasitemia on Day 3 $\geq$ 25 % of count on Day 0.

Late treatment failure (LTF) if the patient develops one of the following conditions during the follow-up period from Day 4 to Day 28, without previously meeting any of the criteria of early treatment failure:

1. Development of danger signs or severe malaria after Day 3 in the presence of parasitemia (same species as on Day 0);

2. Unscheduled return of the patient because of clinical deterioration in the presence of parasitemia

3. Presence of parasitemia on any of the scheduled return on Day 7, Day 14, Day 21 or Day 28 (same species as on Day 0).

Adequate clinical response (ACR) if the patient did not develop any of the criteria of early or late treatment failure before and parasitological clearance has been confirmed throughout the follow-up period.

Interpretation of Test Results: The *in vivo* studies assess the proportion of all treatment failures (ETFs plus LTFs) in the sample of patients included in the study. The statistical procedure adapted for the interpretation of the results allows to test the hypothesis that the proportion of treatment failures is above a certain level in the study area.

A high proportion of ETFs to the first-line antimalarial drug is a strong indicator of the need for changing the first line treatment. In practice, in most situations the proportion of ETFs will not be unacceptably high, and a full investigation with a 14 or 28 day follow-up period is needed to determine the extent of the problem.

A. Subjects:

(1) Number of Subjects: Sufficient volunteers per study site will be enrolled to determine the degree of resistance per site according to the statistical tables in Attachment No. 12. The true rate of resistance in most areas is unknown. We plan on open enrollment

until complete data from several sites within the Upper Amazon Basin are obtained within the limits of personnel and financial resources. Studies may be repeated in the same site on successive years as part of a surveillance program conducted under the DoD Global Emerging Epidemiological Surveillance (GEIS). We plan to complete 3 therapeutic efficacy trials in the 1999 transmission season (Jan-Jul) for an enrollment of about 150-200 people.

(2) Age range and sex: Individuals older than 6 months. No restrictions or recruitment quotas for gender. Known pregnancy is a contraindication to enrollment.

(3) Inclusion criteria:

1. Pf monoinfection with asexual forms
2. Parasite density of greater than 500 per mcl and less than 200 parasites per oil immersion field (MINSA quantification of "4 plus")
3. Age > 6 months
4. Temperature greater than 38C (101F) or history of fever in last 72 hours.
5. Available and willing to return for follow-up

(4) Exclusion criteria:

1. Presence of any of the following "danger" signs or symptoms suggestive of severe malaria:

    Not able to drink or breastfeed
    Repeated vomiting (unable to keep anything down)
    Convulsions during present illness
    Lethargic or unconscious state
    Unable to sit or stand up
    Respiratory distress
    Jaundice (observation) or dark urine (by history)

Severe anemia (Hemoglobin < 5 g / dl)

Hypotension (systolic BP < 80 mm Hg in adults and < 50 mm Hg in children under the age of 5)

2. Presence of another significant illness or chronic disease.

3. Pregnancy (by negative urine pregnancy test no earlier than 48 hours from entry)

4. History of hypersensitvity to medication used in the test.

(5) Source of Volunteers: Volunteers will come from symptomatic patients who seek care at MINSA medical treatment facilities. There will be no active recruitment.

(6) Subject identification: Volunteers entered into the study will be assigned a study code number. Results will be reported in a descriptive form with no links to identifying features of individual patients.

(7) Risks and benefits: Individuals who participate will receive diagnostic examinations and drug therapy for malaria. Clinical and parasitological failures will be more readily identified if they are in the study because volunteers will be followed more closely if they are in the study than they would be if not in the study. Therefore, volunteers may directly benefit by receiving more timely medical care. There will be no cash payments made to volunteers who participate in the study, however folate-free vitamins, toothbrushes, or a small food allotment such as a bag of powdered milk will be given to volunteers as a modest compensation for their time.

There is no unusual risk to those participating in this protocol. There is no unusual risk to those conducting this research. Fingerstick and venipuncture will be performed by qualified phlebotomists. Standard universal safety precautions will be followed when handling all blood samples.

This is a minimal risk protocol which only involves phlebotomy and no significant adverse events are expected. Occasional bruising at the site of venipuncture is the most serious injury that a volunteer can receive by participating in this study. We do not plan to report this as an unexpected adverse event. Malaria is potentially a severe and fatal illness,

particularly in field settings where prompt diagnosis and treatment are not always available. Therefore, unfavorable clinical outcomes may occur in some study volunteers but they are not related to participation in this study. If anything, volunteers are expected to have a decreased risk of severe and complicated malaria if they enroll because they will be followed closer than they would be otherwise.

(8) Special medical care and equipment required: Medical care for volunteers, including medications, will be provided by licensed Peruvian physicians who are also study investigators. All laboratory supplies and required equipment will be provided by US NAMRID or its collaborators. CQ and PS treatment will be given to patients under the supervision of a licensed Peruvian physician according to the dosage regimen outlines in the protocol.

B. Study Design

(1) Tests

a) Weighing - The patient will be weighed on a reliably calibrated scale and recorded to the nearest kg.

b) Measuring body temperatures - Oral temperatures will be measured to one decimal point and reported as degrees fahrenheit with a digital probe thermometer (Filac® electronic thermometer) using a new, disposable probe cover for each measurement. The digital thermometer will be calibrated daily according to the package insert using the manufacturer's calibration probe. Axillary temperatures will be recorded if the patient is too young to cooperate with obtaining an oral temperature. If oral or axillary temperature measurement is less than 36.0°C, the measurement will be repeated.

c) Microscopic smear examination - Preparation and staining of the blood slides follows the procedures outlined in the standard operating procedures (SOPs) of the US NAMRID (see attachment No. 13) using Giemsa staining at pH 7.2. At least three slides will

always be made: one with a thick film (for rapid staining, 10-15 min. with 10% Giemsa stain, and screening while the patient is in attendance), the other two with a thick and thin film on the same slide for subsequent standard staining (45 minutes with 3% Giemsa stain). The slides stained with 3% Giemsa are referred to as "study slides 1 and 2" and will be used for the interpretation of the final result. Microscopy results from the study will be recorded on standard forms (attachment No. 14)

Parasitemia is quantified based on SOPs (see attachement No. 15). The method used depends on whether a white blood cell count (WBC) is available . The same techniques will be employed for establishing parasite counts on each of the subsequent blood film examinations.

A blood slide is considered negative for study purposes when the examination of 200 thick film fields by two indepenedent microscopists (A and B) does not show the presence of asexual forms of *P. falciparum*. The presence of *P. falciparum* gametocytes should be noted irrespective of asexual forms, but the presence of gametocytes alone is not sufficient for enrollment in the study.

d) Hemotological assessment - Hematological assessment will be done by measuring hemoglobin with the Hemocue® system or the QBC Autoread Hematology System, following the receommended manufacturers directions.

e) *In Vitro* culture - Parasites will be cryopreserved according to SOP (see attachment No 16) and transported to NAMRID Lima laboratory for recovery and in vitro culture according to standard methods.

(2) Specimens

a) Amount and schedule of collections: A few drops of capillary whole blood obtained by fingerprick will be used to make the initial smear evaluation. For those with a positive result, venipuncture or a second fingerstick will be performed. Each subject will have a

D0 venipuncture (15 ml). Those subjects who fail therapy will have a second venipuncture (day failure = DF). Therefore each subject will have up to15 to 30 ml drawn in the course of the study depending on whether or not they fail therapy, their age and weight, and their hemodynamic status.

The criterion of a lower age cut-off of 6 months for inclusion in the study is to conform with the WHO/PAHO standard protocol. However, we have found that very young children, less than 3 or 4 years of age, do not tolerate even the frequent fingerpricks (up to 8) required for the study protocol. We will not enroll these patients if both the children and their parents / guardians are not completely supportive. This will enter a very minimal selection bias into the study but we feel it is not significant to affect study endpoints.

Phlebotomy on children will be performed with concern for maintaining adequate blood volume (hemodynamic stability) for perfusion and adequate red cell mass for oxygenation (Hgb per dl). Only children meeting the requirements for hemodynamic stability will be considered for phlebotomy. The standard is in accordance with the age adjusted parameters defining "mild" volume depletion (no more than 3-5% body weight deficit) described in Nelson's Textbook of Pediatrics. Ed. R.E. Behrman, Kliegman R.M. and Arvin. A. M. W. B. Saunders, Philadelphia. In Fluid Therapy, p. 209, In History and Physical Examination, p. 1266, In Diseases of the Blood p. 1383 and 1389. (see attachment No. 19).

Hemodynamic stability will be documented with a systolic blood pressure defined above the 5th percentile for age and sex based on accepted standards reported by the First Task Force On Blood Pressure In Children (Blumenthal, S., et al. Report of the task force on blood pressure in children, Pediatrics, vol. 59, P. 797). The criteria for acceptable pulse are based on published standards for resting heart rate in Nelson's Textbook of Pediatrics. The pulse must be less than the age adjusted, " upper limit of normal." (See attachment No. 19)

If the child meets systolic blood pressure and pulse criteria for hemodynamic stability, adequate red cell volume will then be ensured based on the hemoglobin measurement. A recognized standard of care for critically ill children recommends a minimal hematocrit of 30%, which is equivalent to a hemoglobin of about 10 gm per dl. (Nathan and Oski's Hematology of Infancy and Childhood, 5th Ed. Ed. By David G. Nathan and Stuary H. Orkin, W.B. Saunders Com. Philadelphia, 1998. In Red Cell Transfusion, pp. 1786-1787). However, it is well recognized that clinically stable individuals with chronic anemia tolerate well hematocrits of 20% or less with adequate tissue oxygenation, and hemoglobin levels of 7 to 8 gm/dl are well accepted thresholds for adequate tissue oxygenation in hemodiluted surgical procedures. (Messmer K. Acute preoperative hemodilution; physiological basis and clinical application. In: Tuma RF, White JV, Messmer K, eds. The role of hemodilution in optimal patient care. Munich: W. Zuckschwerdt Verlag, 1989: 54-73 and Fontana JL, Welborn L Mongan PD, et al. Oxygen consumption and cardiovascular function in children during profound intraoperative normovolemic hemodilution. Anesh. Analg 1995;80:219-25.) By meeting the criteria for hemodynamic stability described above, the child, by definition, has a compensated anemia and adequate cardiac output to support oxygen requirements. Therefore, an intermediate hemoglobin value of 8 gm per dl in the presence of hemodynamic stability is set as the threshold for phlebotomy. The volume of blood withdrawn will be limited to 1.0 cc per kg of weight. This represents less than a 2% reduction in red cell mass even with an assumption of 5% volume deficit.

These standards will be incorporated into a standard DRF (attachment No. 19) that will be used on all individuals to determine suitability for phlebotomy. Meeting minimal criteria for phlebotomy and minimal criteria for study participation are different. We may enroll a patient into the therapeutic efficacy trial (meets study inclusion criteria) but not elect to perform phlebotomy on D0.

Each 15 ml sample will be obtained via a needle and syringe from each patient. For samples less than 15 ml, the blood will be divided proportionally as below. The venous blood will be divided as follows:

8.5 ml ACD - 5 ml for cryopreservation for Pf culture, the remainder for DNA preservation in guanidinium HCL

2.5 ml EDTA - blood smears, filter paper, MRDD dipsticks, QBC or Hemocue, IsoCode Stix (for PCR)

The remainder into a 7 ml red top - clot at room temp for at one hour, serum separation. Serum used for clinical chemistries aliquots saved for antibody work.

Results will be available on the same day the slides are made if possible. Slides may be read in the field or at the main laboratory depending on logistical requirements.

b) Evaluations to be made on specimens:

1. Parasite densities will be calculated by counting the number of asexual parasites per µl of blood. If WBC counts are available then quantification in parasites per µl will be obtained by counting the number of asexual parasites per 200 WBCs and multiplying by WBCs per µl. For initial enrollment purposes, a quantification will always be made using 8000 WBCs per mcl to ensure consistency between sites.

2. On selected sensitive and resistant parasites, drug sensitivity profiles will be obtained to a standard panel of antimalarial drugs.

3. DHFR / DHPS genotypes will be determined via mutation specific PCR

c) Quality assurance: No specific measures to assure quality assurance are planned due to funding and logistical considerations. Smears are routinely quality controlled in the NAMRID laboratory. PCR assays and in vitro drug sensitivity assays are not licensed or approved tests and are performed in research laboratories.

d) Specimen storage: Cryopreserved parasites will be stored in vapor phase liquid nitrogen until ready for *in vitro* cultivation. Blood dots intended for PCR assays will be kept in a cool, dark place. Each filter paper is stored in a separate plastic bag with resealable closure and an individual desiccant packet. Each specimen (slide, filter paper, etc.) will be labeled with the study code number, date of collection, and day of study for each subject.

(3) Medications:

a) No investigational medications will be used in these studies.

b) Antimalarial medications: The drugs employed for therapeutic efficacy testing will be of a reliable, quality controlled batch. We plan to purchase drugs from reliable US sources which manufacture in FDA approved facilities to include CQ phosphate (Aralen®, Sanofi Pharmaceuticals) and PS (Fansidar®, Roche Laboratories). Drugs will be stored according to manufacturer's recommendations. The drugs should not be used beyond the expiry date on the package. For eventual identification at a later stage, the manufacturer and batch number of the administered drug will be recorded on attachment No. 10. The following drugs and formulations will be used for the in vivo resistance studies:

<u>1. Chloroquine (CQ)</u>	Tablets 150 mg base	as phosphate or sulphate
	Tablets 100 mg base	as phosphate or sulphate

Treatment with CQ consists of a three-day course with the following doses:

Day-0	10 mg (base)/ kg body weight
Day-1	10 mg (base) / kg body weight
Day-2	5 mg (base) / kg body weight

In case syrup formulations of chloroquine are not available, the bitter taste of

chloroquine may render difficult the administration to infants and young children. This constraint can be overcome by crushing the tablets and mixing them with a little water and sugar on a spoon. For children with a marked tendency of vomiting the crushed tablets can be mixed with banana or other locally available foods.

2. Pyrimethamine / Sulfadoxine (PS)	25 mg P Tablets + 500 mg S

Treatment with PS is given as a single dose equivalent to 1.25 mg pyrimethamine/kg body weight (up to a maximum adult dose of 3 tablets = 75 mg of pyrimethamine). For children the doses appropriate to the various weight groups are shown in Attachment 17.

3. Quinine

Treatment with quinine only is with the oral administration of 30 mg salt / kg body weight per day divided in 3 doses to take every 8 hours. Duration of the treatment will be for seven days. Dose does not exceed 650 mg per dose (two tablets) or more than 6 tablets per day (650 mg X 3 = 1,950 mg). Fractioning of the quinine sulfate tablets for the approximate dose of 30 mg/kg/day is done for children and infants. Patients will receive information about the adverse effects of quinine, especially cinchonism (tinnitus, dizziness and hypoacusia).

4. Quinine - tetracycline

Treatment with quinine plus tetracycline consists of the oral administration of 30 mg/kg quinine divided in 3 doses to take every 8 hours for 7 days. Tetracycline is administered in the dose of 1000 mg per day divided in 2 doses for 7 days. Tetracycline cannot be administered to children less than 8 years old or pregnant women or those who are breast-feeding. Patients will receive information about the adverse effects of quinine, especially cinchonism (tinnitus, dizziness and hypoacusia).

5. Quinine - clindamycin

Treatment with quinine plus clindamycin consists of the oral administration of 30 mg/kg quinine divided in 3 doses to take every 8 hours for 7 days. Clindamycin should be administered in a dose of 20 mg/kg per day divided in 2 doses for 5 days. The patients will receive information about the adverse effects of quinine, especially cinchonism (tinnitus, dizziness and hypoacusia).

c) Other medications:

1. Paracetamol (acetaminophen) is often used to lower temperature and offer symptomatic relief to patients. The effect of paracetamol on parasite clearance time in *Plasmodium falciparum* malaria has been studied (Brandts, et al. Lancet 350:p.704, 1997). This was a randomized trial comparing quinine plus mechanical antipyresis with or without paracetamol in 50 children with uncomplicated Pf malaria in Gabon. The 25 patients assigned to paracetamol received 50 mg/kg per day in suppository form. Fever clearance time (FCT) was shorter (not statistically significant) in the paracetamol group and parasite clearance time (PCT) was longer in the paracetamol (statistically significant) group. In addition, induced concentrations of TNF and production of oxygen radicals was significantly lower in the paracetamol group. The authors conclude that paracetamol has little additional antipyretic effect over mechanical antipyresis, increases PCT, and may actually be clinically detrimental.

Our study population differs form that reported by Brandts, et al. For example, they used relatively high doses of paracetamol (50 mg / kg per day) given rectally. The doses we would use in Peru are in the range of 30 mg / kg / day. Also, the starting parasitemia in the Gabonese children are significantly higher than that

typically found in Peru. In Gabon, the geometric mean was 80,000 parasites per mcl. Using data from 115 patients with a monoinfection with Pf collected from another study (WRAIR No. 687) in Iquitos, Peru, we found a geometric mean of 6,071 parasites per mcl, a significantly lower starting parasitemia. Therefore, we feel that the impact of paracetamol use on our study will be minimal.

The desirability of reducing fever and the best method to do so in persons with malaria are controversial. Past experience with patients with malaria and the parents of children with malaria in Peru have shown that they do expect to receive something for fever and perceive fever as a major problem. Failure to do so may lead them to suspect that the investigators are not taking adequate care of them. Therefore, the option to not use paracetamol for our study subjects in Peru is not desirable.

Use of paracetamol may be a confounding variable and its use should be standardized in our study. We plan to use 3 doses of paracetamol at 10 mg / kg per dose in each subject on entering. The first dose will be given on entry, the second dose about 6-8 hours later and the last dose at 12-18 hours after the first. Parents/guardians may also be instructed to use tepid sponging during the initial 24 to 48 hours. Failure to discuss this issue may lead to perception that treatment is not effective, and the patient (or parents/guardians) may seek alternative medication which may interfere with the study protocol.

2. If during the follow-up, infections other than malaria require the administration of medications with antimalarial activity, e.g. cotrimoxazole or tetracycline, the patient will be excluded from the study. Patients given tetracycline as eye ointment will not be excluded.

3. Chlorpheniramine, an H1 blocker, is sometimes used for the symptomatic relief of pruritus associated with CQ use. However, it has also been shown to reverse CQ resistance in vitro and in vivo. Therefore, chlorpheniramine and other H1 blockers

will be specifically prohibited in this CQ efficacy trial.

d) Alternative treatment of failures:

1. The indication for alternative treatment at any time between Day 0 and Day 28 should be based on both clinical and parasitological criteria. The goal is to prevent a worsening of the clinical condition and prevention of death or progression to complicated malaria. The decision to declare a subject an ETF or LTF and offer alternative treatment is always somewhat subjective. The clinical evaluation on each encounter following initial treatment is documented on attachment No. 12. To the extent possible, the decision to offer alternative treatment will be objective, but the study physician's first priority is the safety of the subject. Therefore, on occasions, some subjects may be offered alternative treatment even though they do not meet criteria listed in attachment No. 12. In those cases, the study physician will list the reasons why the subject was offered alternative treatment.

2. Clinical judgement should always be supported by parasitological evidence (see Classification of Therapeutic Response, above).

3. The recommended alternative antimalarial treatment will be PS in the case of Cq failure and quinine plus tetracycline or clindamycin in the case of poor response to PS. Although the therapeutic efficacy test ends when the patient has been classified as early treatment failure (ETF) or late treatment failure (LTF) and given alternative medication, the study team will ascertain that the alternative medication resolves the clinical illness of the volunteer.

4. If the patient develops any signs of severe or complicated malaria or any of the general danger signs during the follow-up period, he will be referred urgently to the appropriate health facility.

5. All volunteers with documented parasitemia between Day 7 and Day 28,

irrespective of symptoms, will be treated with the alternative antimalarial drug.

(4) Follow-up Procedures:

a. Rarely, if ever, will all patients enrolled for therapeutic efficacy tests complete the posttreatment follow-up. The representativeness of the study diminishes with an increasing number of drop-outs, especially when the reasons of failing posttreatment follow-up are related to an unsatisfactory outcome of the treatment. Therefore, precise documentation of the locations of the volunteers at enrollment, and rigorous tracing of patients who fail to show up on the scheduled days is important to complete data collection. The number of losses will be limited to < 10 %, the maximum permitted dropout rate permitted, if at all possible. The reason for dropping out should be ascertained in every individual case to exclude an association with the outcome of the test.

b. A dropout is defined as a patient lost to follow-up despite fulfilling all inclusion criteria, without developing exclusion criteria during the follow-up period.

c. The following conditions should not be considered as drop-outs but classified as exclusions:

1) Occurrence, during follow-up, of concomitant disease that would interfere with the clear classification of treatment outcome;

2) Movement of a patient from the study site to a place outside the reach of active follow-up (this movement must be unrelated to the response to treatment);

3) Failure to complete the treatment due to withdrawal of consent;

4) Antimalarial treatment administered by a third party during the follow-up period; and,

5) Detection of *P. vivax* malaria during follow-up.

(5) Disposition of Data: A copy of the data sheets and the VAAs will be kept on file by the principal investigator, or at NAMRID after the PI leaves, for at least 10 years after completion of the study. We plan no independent QA audit of the data. We do not plan to complete Volunteer Registry forms (60-R).

(6) Analysis of Data:

a) CRFs will be returned to the PI and data entered into standard data base software programs, by the NAMRID data entry and analysis group. We are currently using the following software:

- Data Entry : Fox-Pro for DOS
- Management databases : Visual Fox-Pro v.5.0
- Analysis : SPSS professional v.7.5 (analysis)
  Epi-Info v.6.04a
  Prism
  EpiCal 2000 v.1.1

b) Statistical Considerations: In trying to estimate the proportion of treatment failures in the overall population from a limited number of patients, bias in the study design is a source of major concern. The results of the study will be biased if its design systematically favors certain outcomes.

Selection bias is likely to occur, where the study subjects are systematically not representative of the patient population at large. For instance, the presence of the medical team may attract those patients who have already suffered a treatment failure with drugs they received earlier or got from elsewhere. On the other hand, the presence of the investigators and the availability of diagnostic and treatment facilities during the study, may attract to the clinic patients who would not have come for treatment

otherwise, especially in remote or highly endemic areas.

Under most circumstances, the investigator will be able to detect major bias in the sample of patients recruited for the study. Close interaction with the local staff in the clinic will be very fruitful to detect these conditions, and the major concerns in this respect should be noted in the activity report. The sample of patients enrolled in the study should be representative of the patients with uncomplicated falciparum malaria seeking treatment in the health services in that locality.

In this respect, a study conducted in an outpatient department (OPD) of a district hospital may provide different results from a study in a Puesto de Salud or in a Centro de Salud, since patients may refer to the hospital only when the clinical condition is severe and serious complications occur. Sometimes patients may have already tried different treatments without success and symptoms were present for a longer period. In some areas the patients visiting the OPD may be highly representative of the resident population in the town itself, and results obtained cannot be extrapolated to the population living in more rural areas.

To a certain extent, it is possible to reduce bias in the survey and to improve the representativeness of the results by following some practical rules. Studies should be carried out in a rural health center very close to a well-defined community, preferably in two or more health centres at relatively short distance. The study team should avoid an awareness campaign in the community and the registration book of the clinic can be used to determine the normal load of patients. Information on drug utilization in the area should be obtained from the clinic staff. Sample analysis will be performed in a blinded fashion, such that laboratory personnel do not have access to clinical data while the tests are being performed.

c) Sample Size Determination: One of the goals of monitoring the therapeutic efficacy of antimalarial drugs is to determine if current MINSA national antimalarial drug policies are still valid, and secondly, to guide the decision to change the recommended treatment

of uncomplicated malaria if a systematic study of a sample of patients shows an unacceptably high proportion of treatment failures. The proportion of clinical failures which is unacceptable can only be considered from within a national program. This will vary with the options and the financial, institutional and personnel resources available to each program.

1. Sample size can be determined using the Lot Quality Assurance Sampling or LQAS method (Lemeshow S and Taber S. Lot quality assurance sampling: single and double sampling plans. World Health Statistics Quarterly. 44:115-132, 1991). This allows identification of communities in which the prevalence of drug resistance is above a predetermined critical level, with smaller sample sizes than would be required using more traditional procedures. Sample sizes may be reduced further while maintaining statistical precision by using the double sampling procedure of LQAS.

2. For calculating the minimal sample size according to this method, the investigators must first define two threshold levels: a level at which the proportion of treatment failures is considered acceptable, and a level of treatment failures which is unacceptable, i.e. above which a change in the first-line drug is indicated.

3. According to the Double Lot Quality Assurance (DLQAS) method the sample size is calculated in two stages, according to the example given below. In the first stage, a relatively small sample is selected and monitored. If the results from the first sample are "extreme" , i.e. very low or very high levels of treatment failures are found, then sampling stops and conclusions can be drawn from the smaller sample. On the other hand, if the results of the preliminary sample are equivocal, then a second sample is chosen and conclusions should be based on the results of both combined samples. If the study shows that the critical proportion of treatment failures is unacceptable according to the threshold defined at the start of the study, then the decision of changing the first-line treatment can be supported. If the proportion of treatment failures is below the threshold level considered as

acceptable, then the area can be maintained under routine monitoring.

4. The size of the sample needed depends on the following parameters:

N = The study population size, presumed to be large;
Po = Upper threshold level of clinical failures beyond which replacement of the drug under study is considered;
Pa = Lower threshold level of clinical failures below which it would be more acceptable to continue the utilization of present drug;
a = Probability of concluding that a community has a low prevalence of clinical failures when, in fact, it has a high level (type I error);
ß = Probability of concluding that a community has a high prevalence of clinical failures when, in fact, it has a low level (type II error).

<u>Example:</u>

a. A prevalence of 25% treatment failures may be considered as an indication for the replacement of the first-line drug. Suppose the health authorities are interested in detecting communities with Po>0.25 and want to be 80% sure (power of the test) that communities with Pa ≤ 0.10 will not be wrongly classified as having high prevalence of drug resistance.

b. It is assumed that the community has a large value of N, and that a = 0.05. Locate in the tables of Attachment 18, the table that correspond to Po = 0.25 and move down the row that correspond to Pa = 0.10. At the first stage of the study, the follow-up of the first 16 (= n1) patients should be evaluated.
If the observed number of treatment failures is 0 (= d1) it can be concluded that the actual proportion of treatment failures in the population of patients consulting with uncomplicated falciparum malaria is significantly less than 25% (Po);

c. If the observed number of treatment failures is greater than 5 (= d2), it can be concluded that the actual proportion of clinical failures in the population is not significantly less than 25 % (Po);

d. If the observed number of treatment failures in the first stage is > 0 and ≤ 5, a second stage of monitoring should be initiated in which more patients are evaluated until either 6 (= d2 + 1) treatment failures have been observed, indicating a high failure rate, or until the total number of patients with complete follow-up has reached 42 (= n1+n2) presenting no more than 5 (= d2) treatment failures, indicating a low prevalence of drug resistance.

5. Rarely, if ever, will all patients enrolled for therapeutic efficacy tests complete the posttreatment follow-up. For this reason, the sample size should be adjusted multiplying the original sample size by the rate of loss to follow-up and exclusion from the study. In practice a minimum of about 20% should be added to the minimal sample size, to allow for drop-outs of patients and for those which will be excluded from the protocol during the follow-up period.

In the example given above the practical approach will be to plan the survey and to recruit patients until 42 (= n1 + n2) are enrolled. The conclusions of the test can already be made if during the follow-up of the first 16 (= n1) patients the number of treatment failures is 0 (= d1) or is greater than 5 (= d2). Once the proper clinical management of the remaining patients has been assured, the resources should be more efficiently used by repeating the survey in a different area.

d) Presentation of the Results:

1. All studies will report general information on the study area, a description of the health facility in which the survey was carried out, the characteristics of the sample population, including information on drug utilization in the area. The frequency of the factors recorded on the CRFs will be presented, and possible bias in the study should be discussed, especially selection bias at the enrollment of the

patients

2. The results should be summarized in a Table indicating the total number of patients enrolled in the study, the number of patients with adequate clinical response, the number of patients with early and late treatment failures, the number of those lost to follow-up, and of those excluded from the study. A detailed account of the reasons for patients lost to follow-up and those excluded from the study should be given. The conclusion of the study should clearly report if the proportion of failures exceeds the "unacceptable upper limit". If the proportion of failures is below the critical level, the area may need to be maintained under surveillance.

3. Correlations of the *in vivo* results with *in vitro* results of resistance obtained via PCR assays or in vitro assays will be secondary endpoints of the study. The prevalence of known mutations in the DHFR and / or DHPS locus correlating to resistance to PS will also be determined if resources permit.

(7) Use of Data: Data will be used to verify degree of resistance to CQ or PS in the study areas and may help in the development of rapid screening assays for CQ resistant parasites. Novel resistant genotypes may be discovered that would enhance theoretical knowledge for resistance. This data may also be helpful to the MINSA in planning rational control strategies for malaria in the Upper Amazon Basin.

9. MODIFICATIONS TO / DEVIATIONS FROM THE PROTOCOL:

A. Modifications to the protocol will be submitted in writing to the scientific review committee and human use review committee (HURC) through the Office of Research Management (ORM) of the WRAIR as appropriate. This protocol will be reviewed initially, continually, and annually by the WRAIR human use review committee.

B. Volunteers will be allowed to withdraw from the study at any time without prejudice or loss of benefits or services to which they are otherwise entitled. Volunteers may be removed

from the study by one of the study physicians if at any time their continued participation could be injurious to their health and well-being. Reasonable deviations form the study protocol will be allowed to account for unforeseen logistic problems that may occur in the study sites.

<u>10. USE OF INFORMATION AND PUBLICATIONS ARISING FROM THIS STUDY:</u> It is anticipated that the results of this study will be presented to the scientific community via oral presentations at meetings and written reports and publications in scientific journals.

<u>11. SPECIAL FUNDING IMPLICATIONS:</u> Funding for this study will be from STO Q, Antiparasitic Drugs, the Military Infectious Disease Research Program (MIDRP) and Global Surveillance.

<u>12. COMPENSATION TO VOLUNTEERS FOR BLOOD DONATION:</u> Volunteers will not be paid for the donation of whole blood required for participation in this study. All volunteers will receive a small food allotment of food items (value less than 3 US dollars) and a 30 day supply of folate free vitamins during the course of study participation.

<u>13. REPORTING OF SERIOUS AND UNEXPECTED ADVERSE EVENTS:</u> Serious and unexpected events adverse experiences will be immediately reported by telephone to the USAMRMC Deputy Chief of Staff for Regulatory Compliance and Quality (302-619-2165) (non-duty hours call 301-619-2165 and send information by facsimile to 301-619-7803). A written report will follow the initial telephone call within 3 working days. Address the written report to the U.S. Army Medical Research and Materiel Command, ATTN: MCMR-RCQ, 504 Scott Street, Fort Detrick, Maryland 21702-5012.

<u>14. SIGNATURE OF PRINCIPAL INVESTIGATOR:</u> I have read the foregoing protocol and agree to conduct the study as outlined herein.

Alan J. Magill MD (signature) 18 Dec 98

Alan J. Magill M.D. Date

14. REFERENCES CITED:

(1965). Resistance of malaria parasites to drugs. Geneva, World Health Organization.

(1967). Chemotherapy of Malaria, World Health Organization.

(1973). Chemotherapy and Resistance to Antimalarials. Geneva, WHO.

Cowman, A. F., M. J. Morry, et al. (1988). "Amino acid changes linked to pyrimethamine resistance in the dihydrofolate reductase-thymidylate synthase gene of Plasmodium falciparum." Proc Natl Acad Sci U S A **85**(23): 9109-13.

We describe the isolation and the sequence of the gene for the bifunctional enzyme dihydrofolate reductase-thymidylate synthase (DHFR-TS; EC 1.5.1.3 and EC 2.1.1.45, respectively) from two pyrimethamine-resistant clones of Plasmodium falciparum, HB3 and 7G8. We have also derived the sequence of the DHFR portion of the gene, by amplification using polymerase chain reaction, for the pyrimethamine-sensitive clone 3D7 and the pyrimethamine-resistant strains V-1, K-1, Csl-2, and Palo-alto. The deduced protein sequence of the resistant DHFR portion of the enzyme from HB3 contained a single amino acid difference from the pyrimethamine-sensitive clone 3D7. It is highly likely that this difference is involved in the mechanism of drug resistance in HB3. The sequence of the DHFR gene from other pyrimethamine-resistant strains contains the same amino acid difference from the sensitive clone 3D7. However, they all differ at one other site that may influence pyrimethamine resistance. The DHFR-TS gene is present as a single copy on chromosome 4 in all pyrimethamine-sensitive and pyrimethamine-resistant isolates tested. Therefore, the molecular basis of pyrimethamine resistance in the parasites tested is not amplification of the DHFR-TS gene.

Desjardins, R. E., C. J. Canfield, et al. (1979). "Quantitative assessment of antimalarial activity in vitro by a semiautomated microdilution technique." Antimicrob Agents Chemother 16(6): 710-8.

A rapid, semiautomated microdilution method was developed for measuring the activity

of potential antimalarial drugs against cultured intraerythrocytic asexual forms of the human malaria parasite Plasmodium falciparum. Microtitration plates were used to prepare serial dilutions of the compounds to be tested. Parasites, obtained from continuous stock cultures, were subcultured in these plates for 42 h. Inhibition of uptake of a radiolabeled nucleic acid precursor by the parasites served as the indicator of antimalarial activity. Results of repeated measurements of activity with chloroquine, quinine, and the investigational new drug mefloquine demonstrated that the method is sensitive and precise. Several additional antimalarial drugs and compounds of interest were tested in vitro, and the results were consistent with available in vivo data. The use of P. falciparum isolates with known susceptibility to antimalarial drugs also permitted evaluation of the cross-resistance potential of each compound tested. The applications and expectations of this new test system within a drug development program are discussed.

Foote, S. J. and A. F. Cowman (1994). "The mode of action and the mechanism of resistance to antimalarial drugs." Acta Trop 56(2-3): 157-71.

The mechanism of action of the antifolate and quinoline antimalarials has been investigated over the last few decades, and recent advances should aid the development of new drugs to combat the increasingly refractile parasite. The molecular description of resistance to the antifolates has been well characterised and is due to structural changes in the target enzymes, but the factors involved in the parasite's ability to circumvent the action of the quinoline antimalarials have yet to be fully elucidated. This review discusses the mode of action of these drugs and the means used by the parasite to defeat our therapeutic ingenuity.

Foote, S. J., D. Galatis, et al. (1990). "Amino acids in the dihydrofolate reductase-thymidylate synthase gene of Plasmodium falciparum involved in cycloguanil resistance differ from those involved in pyrimethamine resistance." Proc Natl Acad Sci U S A 87(8): 3014-7.

Cycloguanil, the active metabolite of the antimalarial drug proguanil, is an inhibitor of dihydrofolate reductase as is another antimalarial, pyrimethamine. Its use has been limited by the rapid development of resistance by parasites around the world. We have determined the cycloguanil- and pyrimethamine-sensitivity status of 10 isolates of Plasmodium falciparum and have sequenced in all these isolates the dihydrofolate reductase (DHFR; 5,6,7,8-

tetrahydrofolate: NADP+ oxidoreductase, EC 1.5.1.3) portion of the DHFR-thymidylate synthase (TS; 5,10-methylenetetrahydrofolate: dUMP C-methyltransferase, EC 2.1.1.45) gene. Instead of the known serine-to-asparagine change at position 108 that is important in pyrimethamine resistance, a serine-to-threonine change at the same position is found in cycloguanil-resistant isolates along with an alanine-to-valine change at position 16. We conclude that pyrimethamine and cycloguanil resistance most commonly involve alternative mutations at the same site. However, we also have identified a parasite with a unique set of changes that results in resistance to both drugs.

Leinicke, T., C. Lucas, et al. (1997). In vivo resistance to pyrimethamine/sulfadoxine for the treatment of Plasmodium falciparum in Peru. 46th Annual Meeting of the American Society of Tropical Medicine and Hygiene, Orlando, FL, USA.

Payne, D. (1987). "Spread of chloroquine resistance in *Plasmodium falciparum*." Parasitology Today 3(8): 241-246.

Peterson, D. S., S. M. Di Santi, et al. (1991). "Prevalence of the dihydrofolate reductase Asn-108 mutation as the basis for pyrimethamine-resistant falciparum malaria in the Brazilian Amazon." Am J Trop Med Hyg 45(4): 492-7.

Pyrimethamine resistance in cultivated laboratory isolates of Plasmodium falciparum is linked to the dihydrofolate reductase mutation Asn-108, a mutation that acts by interrupting drug binding within the active site of the enzyme. To determine the prevalence of this mutation in endemic regions harboring pyrimethamine-resistant malaria, we used a mutation-specific polymerase chain reaction assay to survey P. falciparum strains from a wide section of the Brazilian Amazon. Mutations were identified directly from blood samples without intervening steps of in vitro cultivation. Of 42 samples collected from four states in Brazil, 38 (90%) contained the Asn-108 codon AAC that confers pyrimethamine resistance, four samples contained only the wild-type Ser-108 codon AGC, and none contained the Thr-108 codon ACC found in cycloguanil-resistant pyrimethamine-sensitive strains. These findings indicate that a very high incidence of the Asn-108 DHFR mutation is responsible for pyrimethamine resistance in the Amazon, and they are consistent with recent failure rates reported for

Fansidar (pyrimethamine-sulfadoxine). We suggest that limited use of proguanil be evaluated as an alternative to pyrimethamine.

Peterson, D. S., D. Walliker, et al. (1988). "Evidence that a point mutation in dihydrofolate reductase-thymidylate synthase confers resistance to pyrimethamine in falciparum malaria." Proc Natl Acad Sci U S A 85(23): 9114-8.

Analysis of a genetic cross of Plasmodium falciparum and of independent parasite isolates from Southeast Asia, Africa, and South America indicates that resistance to pyrimethamine, an antifolate used in the treatment of malaria, results from point mutations in the gene encoding dihydrofolate reductase-thymidylate synthase (EC 1.5.1.3 and EC 2.1.1.45, respectively). Parasites having a mutation from Thr-108/Ser-108 to Asn-108 in DHFR-TS are resistant to the drug. The Asn-108 mutation occurs in a region analogous to the C alpha-helix bordering the active site cavity of bacterial, avian, and mammalian enzymes. Additional point mutations (Asn-51 to Ile-51 and Cys-59 to Arg-59) are associated with increased pyrimethamine resistance and also occur at sites expected to border the active site cavity. Analogies with known inhibitor/enzyme structures from other organisms suggest that the point mutations occur where pyrimethamine contacts the enzyme and may act by inhibiting binding of the drug.

Plowe, C. V., A. Djimde, et al. (1995). "Pyrimethamine and proguanil resistance-conferring mutations in Plasmodium falciparum dihydrofolate reductase: polymerase chain reaction methods for surveillance in Africa." Am J Trop Med Hyg 52(6): 565-8.

As chloroquine resistance spreads across Africa, the dihydrofolate reductase (DHFR) inhibitors pyrimethamine and proguanil are being used as alternative first-line drugs for the treatment and prevention of Plasmodium falciparum malaria. Resistance to these drugs is conferred by point mutations in parasite DHFR. These point mutations can be detected by polymerase chain reaction (PCR) assays, but better methods for sample collection, DNA extraction, and a diagnostic PCR are needed to make these assays useful in malaria-endemic areas. Here we report methods for collecting fingerstick blood onto filter paper strips that are air-dried, then stored and transported at room temperature. Cell lysis and DNA extraction are accomplished by boiling in Chelex-100. We also report a nested PCR technique that has improved sensitivity and specificity. These procedures readily detect mixed infections of

parasites with both sensitive and resistant genotypes (confirmed by direct sequencing) and are reliable at parasite densities less than 250/mm3 in field surveys.

Plowe, C. V., A. Djimde, et al. (1996). "Community pyrimethamine-sulfadoxine use and prevalence of resistant Plasmodium falciparum genotypes in Mali: a model for deterring resistance." Am J Trop Med Hyg **55**(5): 467-71.

Pyrimethamine-sulfadoxine (PS, Fansidar; Hoffman-LaRoche, Basel, Switzerland) is now the first-line antimalarial therapy in parts of Africa with high rates of chloroquine-resistant Plasmodium falciparum. With PS resistance increasing and no suitably inexpensive and effective third antimalarial drug available, strategies for delaying the spread of PS resistance in Africa are needed. Community PS usage was measured in two Malian villages, one rural and one periurban, and prevalence of pyrimethamine-resistant P. falciparum genotypes was determined at these sites and two urban sites. The prevalence of resistant genotypes was 22.6% (n = 84) in the periurban village where PS was available from multiple sources and large stocks of PS were observed, and 13.5% (n = 89) and 23.4% (n = 77) in a large town and a city, respectively, where PS is widely available. No pyrimethamine-resistant genotypes (n = 58) were detected in Kolle, a rural village with a community-supported dispensary and clinic where PS is used sparingly and no PS was available in pharmacies or markets. The high rates of pyrimethamine resistant genotypes concurrent with higher PS usage argue for a policy of judicious PS use in Mali and in similar settings. A possible model for slowing the spread of drug-resistant malaria is illustrated by the example of the Kolle clinic.

Plowe, C. V. and T. E. Wellems (1995). "Molecular approaches to the spreading problem of drug resistant malaria." Adv Exp Med Biol **390**: 197-209.

Rieckmann, K. H., G. H. Campbell, et al. (1978). "Drug sensitivity of plasmodium falciparum. An in-vitro microtechnique." Lancet 1(8054): 22-3.

Su, X., L. A. Kirkman, et al. (1997). "Complex polymorphisms in an approximately kDa protein are linked to chloroquine-resistant P. falciparum in Southeast Asia and Africa." Cell **91**(5): 593-603.

Chloroquine resistance in a P. falciparum cross maps as a Mendelian trait to a 36 kb segment of chromosome 7. This segment harbors cg2, a gene encoding a unique approximately 330 kDa protein with complex polymorphisms. A specific set of polymorphisms in 20 chloroquine- resistant parasites from Asia and Africa, in contrast with numerous differences in 21 sensitive parasites, suggests selection of a cg2 allele originating in Indochina over 40 years ago. One chloroquine- sensitive clone exhibited this allele, suggesting another resistance component. South American parasites have cg2 polymorphisms consistent with a separate origin of resistance. CG2 protein is found at the parasite periphery, a site of chloroquine transport, and in association with hemozoin of the digestive vacuole, where chloroquine inhibits heme polymerization.

Wellems, T. E., A. Walker-Jonah, et al. (1991). "Genetic mapping of the chloroquine-resistance locus on Plasmodium falciparum chromosome 7." Proc Natl Acad Sci U S A **88**(8): 3382-6.

The resurgence of malaria in recent decades has been accompanied by the worldwide spread of resistance to chloroquine, a drug once uncontested as the first-line antimalarial agent because of its efficacy and low toxicity. Chloroquine-resistant strains of Plasmodium falciparum counter the drug by expelling it rapidly via an unknown mechanism. In the absence of explicit biochemical knowledge of this efflux mechanism, reverse genetics provides a powerful approach to the molecular basis of chloroquine resistance. Here we report genetic linkage analysis in which 85 restriction fragment length polymorphism markers were used to examine inheritance of the 14 P. falciparum chromosomes in a laboratory cross between a chloroquine-resistant and a chloroquine-sensitive parasite. Inheritance data from 16 independent recombinant progeny show that the rapid efflux, chloroquine-resistant phenotype is governed by a single locus within an approximately 400-kilobase region of chromosome 7. Identification and characterization of genes within this region should lead to an understanding of the chloroquine-resistance mechanism.

Zolg, J. W., J. R. Plitt, et al. (1989). "Point mutations in the dihydrofolate reductase-thymidylate synthase gene as the molecular basis for pyrimethamine resistance in Plasmodium falciparum." Mol Biochem Parasitol **36**(3): 253-62.

The dihydrofolate reductase-thymidylate synthase (DHFR-TS) bifunctional complex from pyrimethamine-sensitive (3D7) and drug-resistant (HB3 and 7G8) clones from Plasmodium falciparum was purified to homogeneity. A modified sequence of purification steps with a 10-formylfolate affinity column at its center, allows the isolation of the enzyme complex with a 10-fold higher yield than previously reported, irrespective of the pyrimethamine resistance of the parasites. Titration of the homogenous DHFR-TS complex with the inhibitor revealed a 500-fold lower affinity of the enzyme from clone 7G8 for the drug than found with the enzyme from clone 3D7. Direct comparison of the homogenous enzyme preparations on SDS-PAGE revealed no difference in the molecular mass of the DHFR-TS from the 3 clones, nor could a reproducible difference be detected in the peptide patterns obtained after digesting the DHFR-TS complex with various proteases. The amplification of segments from the DHFR-TS coding region of the 3 clones and 7 isolates of P. falciparum by polymerase chain reaction resulted in fragments of the predicted length without any size heterogeneity. The DNA sequence of the DHFR coding region from FCR-3, 3D7, HB3 and 7G8 differs in a total of 4 nucleotides. One point mutation changes amino acid residue 108 from threonine (FCR-3) or serine (3D7) to asparagine (HB3 and 7G8). The presence of asparagine-108 appears to be the molecular basis of pyrimethamine resistance of HB3 and 7G8. The degree of resistance is associated with a point mutation affecting the codon for amino acid 51 in 7G8.

WRAIR No. 719,
HSPD Log No. A-8568
CQ/PS-R in the Amazon

Attachment No. 1
CQ/PS-R Pf Time and Event

Key Time Points of Study (days)	DO	D1	D2	D3	D7	D14	D21	D28	D symp	DR/ETF/LTF
Clinic visit	1	2	3	4	6	7	8	9		
Clinical Evaluation	X	X	X	X	X	X	X	X	X	X
Measuring oral / axillary temperature	X	X	X	X	X	X	X	X	X	X
Weighing patient	X									
Attach No. 2- Inclusion / Exclusion	X									
Attach No. 3 - Informed Consent	X									
Attach No. 4 - Subject Info form	X									
Attach No. 5 - CQ Rx confirmation	X	X	X							
Attach No. 11- PS Rx confirmation	X									
Attach No. 12 - Post Rx f / u		X	X	X	X	X	X	X	X	X
Parasitological Assessment (thick / thin smear)	X	X	X	X	X	X	X	X	X	X
Attach No. 10 - MRDD performance	X	X	X	X	X	X	X	X	X	X
Venipuncture	X									X
Rx #1 CQ - 10 mg base / kg	X									
Rx #2 CQ - 10 mg base / kg		X								
Rx #3 CQ - 5 mg base / kg			X							
Rx PS	X									
QBC or Hgb	X							X		
Blood volume	15									15
Blood volume (cumulative)	15									30
Alternative Rx		X**	X**	X**	X**	X**			X**	X
X** = alternative Rx										

44

FLOW SHEET FOR TESTING

Initial Evaluation

Symptomatic patients present to MINSA facility → Fingerprick positive for Pf monoinfection → Interviewed by study personnel for inclusion/ exclusion criteria. (attach No. 2) → Counseling about study Sign informed consent (attach No. 3)

Study personnel complete Attach No. 4 → DO venipuncture attach No. 18 → DOT (attach No. 10 for CQ) DOT (attach No. 11 for PS)

Continuing Evaluation

Volunteers return to MINSA for scheduled follow-up or symptomatic → DOT (days 2 and 3 for CQ) Fingerprick per timeline Clinical evaluation (attach No. 12)

Failure Evaluation

Clinical Failure (ETF, LTF) or Parasitolgic failure (RII, RII) → DF Venipuncture → Insure adequate clinical care / follow-up

Attachment 2: Inclusion / Exclusion Criteria Date:__ __ / __ __ / __ __
Study No. 719, HSPD Log No. A-8568 D D M M Y Y

Inclusion Criteria:

1. Pf mono-infection with asexual forms	YES	NO
2. Parasite density greater than 500 parasites per mcl but less than 200 parasites per oil immersion field (MINSA "4 plus")	YES	NO
3. Age > 6 months	YES	NO
4. Temperature greater then 38C (101F)	YES	NO
5. History of fever in last 72 hours	YES	NO
6. Available and willing for follow-up	YES	NO

If the volunteer has any of the above inclusion criteria circled no, he is not eligible for the study. Patient will be referred to clinical point of care for further evalauation.

Exclusion Criteria:

1. Does the patient have any of the following signs or symptoms of severe malaria?

Not able to drink or breastfeed	YES	NO
Repeated vomiting (unable to keep anything down)	YES	NO
Convulsions during present illness	YES	NO
Lethargic or unconscious state	YES	NO
Unable to sit or stand up	YES	NO
Respiratory distress	YES	NO
Jaundice or icterus (observation)	YES	NO
Severe anemia (Hemoglobin < 5.0 g/dl)	YES	NO
Hypotension (systolic BP < 80 mm Hg in adults and < 50 mm Hg in children under the age of 5)	YES	NO
2. Presence of another significant illness or chronic disease?	YES	NO
3. Pregnancy (negative urine pregnancy test)?	YES	NO
4. History of hypersensitvity to medication used in the test?	YES	NO

If the volunteer has any of the above exclusion criteria circled yes, he is excluded and not eligible for the study. Patient will be referred to clinical point of care for further evaluation.

•**Is patient willing to sign informed consent?**	**YES**	**NO**
•**Is this volunteer eligible for the study?**	**YES**	**NO**

Signature of responsible investigator: ______________________________

Printed name of responsible investigator: ______________________________

Attachment 3: Drug Resistant Malaria in Peru
Volunteer Informed Consent Agreement (English), Page 1 of 2
WRAIR Protocol No. 719, HSPD Log No. A-8568 (version 15 Dec 98)

1. You are being asked to volunteer for a research study that will see how well the current medications for the treatment of malaria are working in Peru. You have falciparum malaria, a potentially serious and debilitating illness. You have been chosen for this study because you live in the local area, and are available for follow-up over the next 28 days. You and about 50 volunteer patients from this area will be included in this study. Your participation in the study will include taking the medications prescribed, donating a blood sample, and coming back to the health post on at least eight occasions over 28 days.

2. This medical research project is entitled, "Resistance to chloroquine (CQ) and pyrimethamine-sulfadoxine (PS) for the treatment of uncomplicated *P. falciparum* malaria in the Upper Amazon Basin. ". The project is being conducted in Peru under the direction of the principal investigator, Alan J. Magill, M.D., LTC, U.S. Army and Dr. Alejandro Llanos, Director of the School of Public Health of the Universidad Cayetano Peruana Heredia (UPCH) in Lima, Peru. The study will be conducted with the collaboration of the regional and local Peruvian Ministry of Health (MINSA).

3. The procedure for this study has been explained to you as follows: You will be interviewed by a study physician, who will ask questions about your past medical history related to malaria and your current illness. MINSA workers will be examine your blood to look for malaria parasites, just the same as if you were not participating in this study. If you meet the inclusion and exclusion criteria for the study you will be asked to participate. You will take the medications given to you by the doctors to treat your malaria.

4. Between 10 and 15 milliliters of blood, about two to three teaspoons, will be drawn from a vein in your arm by a needle when you are included. The blood drawing will be performed by experienced health care personnel of the US Naval Medical Research Detachment (US NAMRID), Universidad Peruana Cayetano Heredia (UPCH) or the MINSA who are conducting this study.

5. There is no risk to you or your children, if they were chosen for the study, from participating in this study other than the discomfort of having a needlestick. Your arm will be cleaned with alcohol, and new sterile needles will be used to minimize the risk of infection. If you have any discomfort, you will receive medical treatment and be observed until you feel better. You will benefit from participating in this study because you will be closely followed over the next 28 days. If you continue to suffer from malaria, you will receive an alternative treatment, quinine plus clindamycin or quinine plus tetracycline according to MINSA guidelines, which will treat the illness. There will be someone at the health post every day so that, even on days between scheduled visits and on weekends you may come in for a checkup if you feel ill. Information about your illness, related to malaria, obtained during the study will be shared with clinicians caring for me in an effort to improve the accuracy of diagnosis and efficacy of therapy available to you. Also, you will receive a small provision of food or snacks, like all other participants. There is no cost to you for participating in this study.

6. If you have any questions about your participation in this research study, you may contact the local MINSA representative, study personnel or Dr. Alan J. Magill of the US NAMRID at 01-561-2733 / 3043. If you have any questions about your rights as a participant in this study, you may direct them to Dr. Pedro Legua, the medical monitor, at Universidad Peruana Cayetano Heredia at 01-482-3903 / 3910.

7. Your participation in this study is voluntary and if you decide not to participate it will not prejudice or interfere in your medical care and treatment.

8. The MINSA workers or the study investigator's are responsible for the laboratory testing of the blood sample which will be used to determine my diagnosis, your care and treatment. US NAMRID or the Walter Reed Institute laboratories or their designees are responsible for the research testing of my specimen, but their research results will not be used to guide my care.

9. Medical inspectors from both the United States and Peru who are checking to be certain that this research is performed in a safe, legal, and approved manner may be allowed to directly inspect the forms which include information about you, and information about your test results. Representatives of the collaborating institutions are eligible to review research records as a part of their responsibility to protect human subjects in research. You agree to allow such inspection. However, in all publications, reports and presentations resulting from this research study, my name will not be used. In addition, a sample of your blood (or serum) can be kept for use in future studies.

10. Although you will not be notified about the results of this study, local MINSA members will receive a report of the results when the study is completed. The researchers conducting the study intend to publish its results in medical journals.

11. The original copy of this consent form will be retained by the principal investigator. I certify that I have received a copy of this consent form and understand that signing this form verifies my willingness to voluntarily participate in the study.

•Adult (18 years of age or older): Yes [ ] No [ ]
Volunteer signature: ______________________ Date: ____________
Volunteer printed name: ______________________________________
Last Name (Paternal) Last Name (Maternal) First Name
Witness signature: ______________________ Date: ____________

•Child or Minor (Less than 18 years): Yes [ ] No [ ]
Parent / Protector signature: ______________________________
Parent / Protector printed name: ____________________________
Last Name (Paternal) Last Name (Maternal) First Name
•Investigator signature: ______________________ Date: ____________

Date: __ __/__ __/__ __
D D M M YR

Study No:

•Baseline Demographics

Nombre:	Apellido Paterno:	Apellido Materno:

Age: ____________ Sex (circle one): Male Female

Current location: ______________________________ (community)

What is the name of your local health post (Puesto de Salud)? ____________

What is the name of your local Central health post (Centro de Salud)? ____________

•Baseline Malaria Data

Number of episodes of smear positive malaria in 1998? 0 1 2 3 > 3 ____ don't know

Date of last episode of smear positive malaria? ____________

Species causing last episode? falcip vivax other don't know

Number of illnesses attributed to malaria in 1998? ____________

Date of last drug therapy for malaria? Date: __ __/__ __/__ __ don't know
D D M M YR

Treatment received? CQ PQ PS quinine TCN

Other: ____________ don't know ____________

•Current Symptoms

Feeling ill for how many days prior to coming to clinic? 1 2 3 4 5 6 7

Symptoms: Headache Low back ache Vomiting Chills Rigors

Currently taking antimalarial drugs? YES NO

If YES, what drugs? chloroquine primaquine PS quinine TCN

Other: ____________

How long? ____________ Dose? ____________

- **Physical Examination**

Temperature: ______________ C or F RR rate: ______________ / min

BP: ______ systolic / __________ diastolic Pulse: ______________ / min

Spleen size (Hackett score): 0 1 2 3 4 5

Comment:

•Laboratory:

1. Smear obtained	YES NO		2. Urine Pregnancy test	POS	NEG
3. Optimal	Done	Not done	4. Parsight F	Done	Not done
4. Hemoglobin testing?	YES	NO	6. Other testing performed?	YES	NO
			If YES, what tests? ______________		

- **Does the subject have any "danger signs" that require removing patient from the study and consideration of alternative therapy?**

Persistant vomiting	YES	NO
Seizures	YES	NO
Glasgow score of less than 15 for adults or 5 for children	YES	NO
If YES, what is score	________(scale provided to study investigators)	
Janudice or icterus apparent	YES	NO
Severe dehydration		
"tenting" of skin	YES	NO
sunken eyes	YES	NO
Respiratory Difficulty		
Use of accessory muscles	YES	NO
RR > 50 for age < 12 months	YES	NO
RR >40 for age 12-59 months	YES	NO
RR > 30 for age > 60 months	YES	NO
Hypotension		
< 80 mm Hg systolic in adults	YES	NO
< 50 mm Hg systolic in children	YES	NO

Investigator signature block: ______________________________ Date: __________

Procedure for aseptic "blood culture"venipuncure:

1. Antecubital space of either right or left arm will be cleaned with a bactericidal wipe.
2. Area above antecubital vein will then be wiped clean with an alcohol pad.
3. Area will then be coated with betadine antiseptic and allowed to set for 2-3 minutes.
4. Vein to be used is then swiped clean with an alcohol pad and allowed to air dry
5. Phlebotamist then inserts a new, disposable 20 or 21 g needle attached to a Vacutainer hub in a vein without touching the vein with gloved hands.
6. Three blood tubes are filled with venous blood: one 7.5 ml ACD blood tube, one 5 ml red top tube, and one 2.5 ml EDTA blood tube.

OptiMAL®
Operating Instructions and Test Interpretation Instructions

OPERATING INSTRUCTIONS

1. Refrigerate buffers and unopened test strips

2. Tests are sensitive to humidity: Allow package to come to room temperature before opening

3. Remove test strip and immediately reclose container tightly. Do NOT return to refrigerator after opening

4. Using disposable 10 mcl pipette from OptiMAL kit, draw up 10 mcl blood from tube of patient's blood or from fingerstick

5. Dispense two (2) drops (30 mcl) Buffer A into test well

6. Add 10 mcl undiluted whole blood to test well, and mix well

7. Place one OptiMAL® test strip, thin end down into well, into test well. Allow all of sample to wick completely up the test strip (this takes 8-15 minutes)

8. Dispense four (4) drops of Buffer B into a second, clean, test well

9. If complete wicking of patient's sample onto test strip has not occurred by 15 minutes, proceed to step 10 anyway

10. Transfer the test strip to the second well containing Buffer B, placing thin end down into well. Read test strip after blood color has cleared (approximately 2 minutes), viewing results under direct or indirect sunlight

11. Refrigerate Buffers A and B when not in use

OptiMAL®

Operating Instructions and Test Interpretation Instructions

INTERPRETATION INSTRUCTIONS

1. Positive: Two or three colored bands should appear across the white central area. The top band is a positive control. Bands will be read as positive if present, no matter how faint.

   a. Three bands indicate *P. falciparum*

   b. Two bands indicate *P. vivax, P. ovale,* or *P. malariae*

2. Negative: Only one colored band at the top of the white central area

3. Invalid: No colored band at the top.

   Result should be discarded and test repeated.

4. Illustration of possible test results:

Negative | Positive, *P. falciparum* | Positive *P. vivax, P. ovale* or *P. malariae* | Invalid

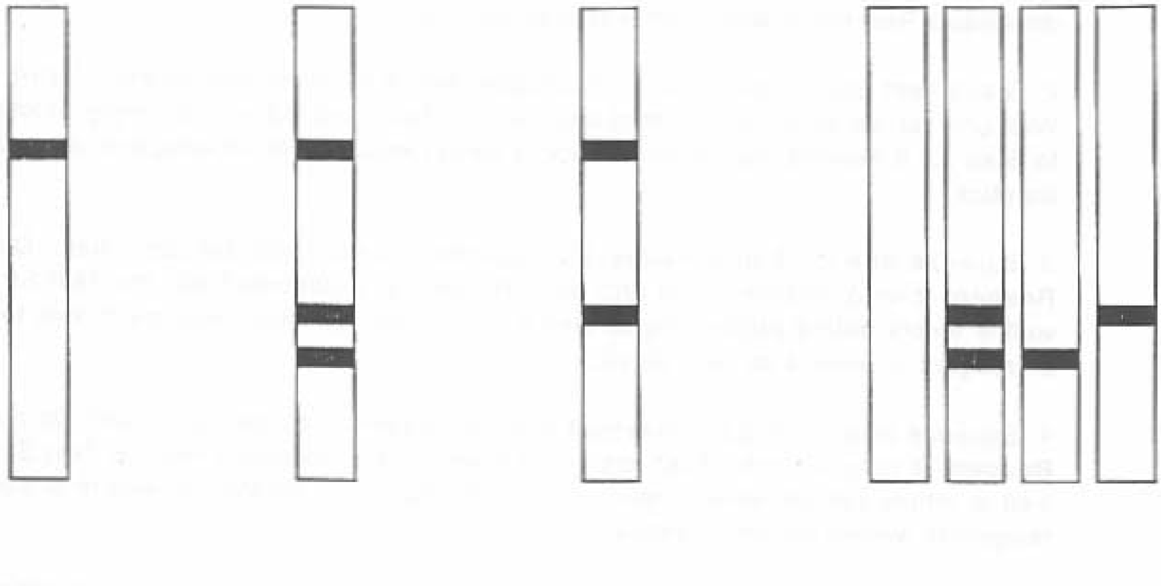

SPECIMEN PREPARATION:

Place one 10-well card in Reaction Stand. Discard card after use.

1. Squeeze **three** (3) drops of **Reagent 1** (lysing agent) into a **DispensTube®** device. Stand **DispensTube®** device in Reaction Stand.

2. Fill capillary tube, from end **farthest** from line, directly from a collection tube of well mixed venous blood, or from a finger or heel puncture. Fill tube by capillary action to line.

3. Keep tube nearly horizontal and roll between the fingers several times to mix the blood with the anticoagulant coating.

4. Drain the blood from the capillary tube into the **DispensTube®.** If necessary, use a rubber bulb to force blood from the capillary tube. **Do not mouth pipette.** Insert the empty end of capillary tube into the small opening of the bulb. Holding the large opening of the bulb closed with the index finger, then squeeze the bulb. Discard used capillary tube into biohazard sharps container.

5. Place a **DispensTube®** tip onto the **DispensTube®.** DO NOT INVERT THE TUBE UNTIL READY TO DISPENSE.

TEST DEVELOPMENT

Remove Test Strip from vial just prior to use. Recap vial after removing Test Strip. Label Test Strip with patient identification label over gray area.

1. Squeeze **one** drop of **whole lysed blood** from the **DispensTube®** into one well in the disposable Reaction Stand. Do not drop blood onto dipstick.

2. Stand Test Strip in the drop of lysed blood with the patient identification facing forward. Wait until all the blood is absorbed into the Test Strip and the well is empty before proceeding to Step 3. If needed, reposition dipstick in well to ensure that all sample is wicked up onto dipstick.

3. Squeeze **one** (1) drop of **Reagent 2** (detection reagent) into the same well. Do not drop **Reagent 2** onto dipstick. Wait until all of Reagent 2 is absorbed into the Test Strip and the well is empty before proceeding to Step 4. If needed, reposition dipstick in well to ensure that all reagent is wicked up onto dipstick.

4. Squeeze **two** (2) drops of **Reagent 3** (wash reagent) into the same well. Do not drop **Reagent 3** onto dipstick. Wait until all of Reagent 3 is absorbed into the Test Strip and the well is empty before reading result. If needed, reposition dipstick in well to ensure that all reagent is wicked up onto dipstick.

RESULTS: Read immediately in well-lighted area, viewing under direct or indirect sunlight

Attachment 8: Optimal MRDD DRF
WRAIR protocol No. 719 **Patient ID Number:**___________

SITE:
Kit lot No. : Date:__ __ / __ __ / 98
DD MM YY

Test performed by (initials): __________

Test Interpretation by (initials): __________ Photostandards used: YES NO

•Control Line present? 0. NO 1. YES

Intensity: 0.25 0.5 1 2 3 4

Comments:

•Test Line No. 1 present? 0. NO 1. YES

Intensity: 0.25 0.5 1 2 3 4

Comments:

•Test Line No. 2 present? 0. NO 1. YES

Intensity: 0.25 0.5 1 2 3 4

Comments:

•Background appearance: 1. white 2. Streaked 3. Dark / stained

Does colored background affect interpretation of test result: 0. NO 1. YES

Comment:

•Test Result: NEG Plasmodium, not Pf Pf or mixed Not valid

Completed:	Signature/initials:	__________	Date:	__________
Investigator review:	Signature/initials:	__________	Date:	__________
Data entered #1:	Signature/initials:	__________	Date:	__________
Data entered #2:	Signature/initials:	__________	Date:	__________

Attachment 9: Parasight™ F MRDD DRF
WRAIR protocol No. 719 **Patient ID Number:** ____________

SITE:
Kit lot No. : Date:__ __ / __ __ / 98
DD MM YY

Test performed by (initials): ________

Test Interpretation by (initials): ________ Photostandards used: YES NO

•Pf Control Dashes present? 1. YES 2. NO

Intensity: 0.25 0.5 1 2 3 4

Comments:

•Pv Control Dashes present? 1. YES 2. NO

Intensity: 0.25 0.5 1 2 3 4

Comments:

•Pf Test Line present? 1. YES 2. NO

Intensity: 0.25 0.5 1 2 3 4

Comments:

•Pv Test Line present? 1. YES 2. NO

Intensity: 0.25 0.5 1 2 3 4

Comments:

•Background appearance: 1. White / clean 2. Streaked 3. Dark / stained

Does colored background affect interpretation of test result: 0. NO 1. YES

Comment:

•Test Result: 1. Negative 2. Positive, P. Falciparum 3. Uninterpretable

Completed:	Signature/initials:	________	Date:	________
Investigator review:	Signature/initials:	________	Date:	________
Data entered #1:	Signature/initials:	________	Date:	________
Data entered #2:	Signature/initials:	________	Date:	________

Attachment 10: Confirmation of Administration of CQ Therapy
WRAIR Protocol No. 719, CQ/PS resistance in the Amazon

Study Number: __________ Weight________(kg) D0 Date:__ __ / __ __ / 99
D D M M

Investigator: Solari Other: ______________________

•Dose Calculation: 25 mg (base)/ kg CQ = 25 X ________ = __________ mg / kg total dose

D0 dose = 10 mg / kg = total dose / 2.5 = ______________ 2nd investigator initials: ___

D1 dose = 10 mg / kg = total dose / 2.5 = ______________ 2nd investigator initials: ___

D2 dose = 5 mg / kg = total dose / 5 = ______________ 2nd investigator initials: ___

•Dose prescribed in 150 mg (base) tablets

D0 dose = __________ tablets 2nd investigator initials: _________

D1 dose = __________ tablets 2nd investigator initials: _________

D2 dose = __________ tablets 2nd investigator initials: _________

•Certification of Tablets Being Provided to Patient

DO: ________ tablets given to volunteer D0 Date:__ __ / __ __ / 99

Tablets swallowed YES NO Witness initials: ______ Subject initials: __

Vomiting within 30 minutes YES NO Witness initials: ______ Subject initials: __

D1: ________ tablets given to volunteer D1 Date:__ __ / __ __ / 99

Tablets swallowed YES NO Witness initials: ______ Subject initials: __

Vomiting within 30 minutes YES NO Witness initials: ______ Subject initials: __

D2: ________ tablets given to volunteer D2 Date:__ __ / __ __ / 99

Tablets swallowed YES NO Witness initials: ______ Subject initials: __

Vomiting within 30 minutes YES NO Witness initials: ______ Subject initials: __

Study Number: ____________ Weight________(kg) D0 Date:__ __/__ __/ 98
D D M M

Investigator: Solari Other: ______________________

•Dose Calculation:

Weight in kg ____________ X 1.25 mg P = __________ mg P required (not to exceed 75 mg)

D0 dose = 1.25 mg / kg = ________________ 2nd investigator initials: __________

•Dose prescribed in tablets with 25 mg pyrimethamine (P) plus 500 mg of sulfadoxine (S)

mg P divided by 25 mg per tablet = _________ mg P / 25 mg Pper tablet = ______ tablets
(to nearest quarter)

D0 dose = ___________ tablets 2nd investigator initials: __________

•Certification of Tablets Being Provided to Patient

DO: _________ tablets given to volunteer D0 Date:__ __/__ __/ 99

Tablets swallowed YES NO Witness initials: _______ Subject initials: __

Vomiting within 30 minutes YES NO Witness initials: _______ Subject initials: __

Subject Number: ________ Date: __ __ / __ __ / 99 DAY ____ OF TEST
D D M M

•Current Condition

	Circle Response:		
Does the patient feel ill now?	YES	NO	
Has the patient been ill since last visited?	YES	NO	
Does the patient feel better, worse, or the same since last seen?	Better	Worse	Same

•If the patient is symptomatic, ask the patient if he or she has any of the following symptoms (circle response)

Fever	YES	NO	Headache	YES	NO
Malaise	YES	NO	Vomiting	YES	NO
Nausea	YES	NO	Diarrhea	YES	NO
Muscle pain	YES	NO			

Went to School/Work	YES	NO
Stayed at Home	YES	NO
Stayed in Bed	YES	NO

• Physical Examination

Temperature: ________ C or F RR rate: ________ / min

BP: ____ systolic / ______ diastolic Pulse: ________ / min

Comment:

•Laboratory:

1. Smear obtained	YES	NO
2. Optimal	Done	Not done
3. Parsight F	Done	Not done
4. Other testing performed?	YES	NO

If YES, what tests? ____________________

• **Does the subject have any "danger signs" that require removing patient from the study and consideration of alternative therapy?**

	Circle Response:	
Persistant vomiting	YES	NO
Seizures	YES	NO
Glasgow score of less than 15 for adults or 5 for children	YES	NO
If YES, what is score	________(scale provided to study investigators)	
Janudice or icterus apparent	YES	NO
Severe dehydration		
"tenting" of skin	YES	NO
sunken eyes	YES	NO
Respiratory Difficulty		
Use of accessory muscles	YES	NO
RR > 50 for age < 12 months	YES	NO
RR >40 for age 12-59 months	YES	NO
RR > 30 for age > 60 months	YES	NO
Hypotension		
< 80 mm Hg systolic in adults	YES	NO
< 50 mm Hg systolic in children	YES	NO

If any of the above "danger signs" are marked YES, the patient requires urgent evaluation by a study physician and alternative therapy for malaria or another condition as appropriate. The patient will be removed from the study. Final classification of ETF, LTF, or exclusion will depend on outcome of clinical and laboratory evaluation. If all responses above are marked NO then proceed:

•Continued participation of the subject in the study is: Acceptable Not-acceptable

If not acceptable, is subject an ETF LTF Dropout Exclusion

Investigator signature: ______________________

A. Blood Smear Slide Preparation

1. Use frosted edge, pre-cleaned glass microscope slides. If pre-cleaned slides are not available, clean slides with 70% ethyl alcohol and let air dry.

2. Make two slides per patient. Label each frosted end of the slide with study information as required per protocol using pencil or pre-printed study labels.

3. Make a thick and a thin blood smear on every slide.

4. Verify that blood is being placed on the correct side of the frosted end slides visually or by feel. Using the pipette, with filter-type tip, place a 10 mcl drop of blood close to the frosted edge of the slide for the **thick** smear and a 4 mcl drop close to thick smear for the **thin** smear.

5. To make the **thin** blood smear, spread the small (4 mcl) drop of blood to the far end of the slide with a second slide. The edge of a clean slide is placed at 30-45 degree angle in front of the drop of blood, pulled back into the drop, and held until the suspension is spread along at least two thirds of the width of the "spreader" slide. It is then pushed forward in a smooth, continuous motion. A properly prepared thin film is thick at the beginning end and thin or "feathered" at the other end. The feathered end of the smear should not reach to the end of the glass slide. The feathered end should have areas optimal for microscopy that are only one cell layer thick. The thin smear is best prepared immediately after applying the drop of blood, before any drying occurs.

6. To make a **thick** blood smear, use the corner of the same slide used to make the thin smear and mix and spread the larger drop (6 mcl) of blood over a circular area about 1 cm (about the size of a dime) in diameter. Continue stirring gently for a few moments and then lift the corner out of the center of the smear, trying not to leave any bubbles in the smear. If bubbles are seen, stir again with the corner of the slide until no bubbles remain, and/or break the bubbles with the sharp corner of the spreading slide.

7. Let the slides dry in a horizontal position. Drying can be aided by letting warm air from a blow dryer pass over the thick smear for 15-20 minutes. A dry smear can easily be recognized by holding to the light and noting any wet areas. Dry slides can be stored vertically in slide boxes. Thick blood smears should dry for at least one hour.

8. After application of blood smear, both during and after drying, slides should be protected from exposure to insects. An acceptable method is placement of slides flat within a flat box or tray with an overlying screen or mesh panel, the panel sufficiently elevated over the slides as not to touch them. Other locally determined methods which prevent insect contact while permitting blood smear drying may be used. The method used should be documented.

B. Blood Smear Slide Staining

1. Slides must dried be thoroughly. Thick blood smears should dry for at least one hour (can be dried more rapidly, in 10 minutes, using warm air blower, if warranted for clinical urgency).

2. Hold the slide in a slanted position with the **thin film down** and cover the **thin film** with a few drops of absolute methyl alcohol. Be sure **NOT** to get alcohol fumes on the thick films. Alternately, one can dip the thin film **briefly** in a container of methyl alcohol for fixing the thin film, taking great care not to "fix" the thick film with the vapor of the alcohol.

3. Let dry in a vertical position with the **thick film up.** Be sure the slide is thoroughly dry before staining. The introduction of even a minute amount of methyl alcohol into the stain dilution will interfere with the dehemoglobinization of the thick film.

4. Stain in 3% Giemsa for 45 minutes.

5. Rinse the slide briefly by dipping slide in and out of a jar or beaker of buffered water.

6. Let dry in a vertical position.

7. After staining, during drying, slides should be protected from exposure to insects. An acceptable method is placement of slides within a box with an overlying screen or mesh panel, the slides so arranged that the stained smear is not touched by the panel. Other locally determined methods which exclude insect contact while permitting stain drying may be used. The method used should be documented.

C.Preparation of Giemsa Stain

Ingredients:

Giemsa stain concentrate (EM Sciences, stock number GX0085/5)
Triton buffered water

Procedure:

1. Using a clean, dry pipette, remove 1.5 ml of Giemsa stain concentrate from bottle. No buffer or aqueous solution is introduced into bottle containing the Giemsa concentrate.

2. Add 1.5 ml of Giemsa concentrate to 48.5 ml of Triton-buffered water solution.

3. Filter before use. Store in a brown glass bottle. An empty, dry methyl alcohol bottle is ideal. Never put a wet or used pipette in the stain. Preferably, pour a quantity of stain from the stock bottle into a small bottle for current use.

D. Preparation of Triton Buffered Water

1. Alkaline Buffer

Na2HPO4 ....9.5 g
Distilled water, to make ....1000.0 ml

2. Acid Buffer

NaH2PO4 ....8.0 g
Distilled water, to make ....1000.0 ml

The acid and alkaline buffers may be kept in separate glass stoppered bottles for long periods.

3. Buffered water

a. Ingredients:

Acid Buffer (NaH_2PO_4) ....39 ml
Alkaline Buffer (Na_2HPO_4) ....61 ml
Distilled water ....900 ml

b. Add buffer solutions to distilled water and mix thoroughly. The pH should be 7.0 to 7.2 for satisfactory stain results. If the pH is above or below this range, it can be adjusted by the addition of the proper buffer.

4. Triton X-100 is moderately viscous and for routine laboratory use, a **10% aqueous dilution** is prepared. This solution will keep indefinitely if tightly stoppered.

5. Add 1 ml of the stock 10% aqueous dilution of Triton X-100 to 1000 ml buffered water. The concentration of Triton will be 0.01%.

E. Blood Smear Interpretation

1. Accurate microscopy is absolutely essential.

2. In general, at least two slides are prepared from each encounter with the patient. The slides are coded with a study No., site code, subject code number, date, and time. One slide is stained in the field and read as soon as possible, the second is saved to be stained in a more controlled setting.

3. Each slide will be read by two "certified" NAMRID microscopists. A certified NAMRID microscopist is one in whom a proficiency set of slides has been accurately interpreted. The certified microscopists, Microscopist A and microscopist B, will then record their interpretations on attachment 14.

4. Examine films using bright field microscopy at 1000x magnification under oil immersion. Microscopy for this study will be performed using Olympus model CH-2 microscopes, using 10x type WHK 10x/20L or WHK 10x/20L-H eyepieces and 100x oil type DPlan 100 1.25 oil 160/0.17 objectives.

5. Blood smear interpretation will be performed by two blinded experienced microscopists. Microscopist A will read 200 thick film fields of Slide #1 and render a smear interpretation (Positive or Negative for asexual *Plasmodia* forms; gametocytes alone will NOT cause a slide to be classified positive). Results will be recorded on the appropriate DRF. Microscopist B will also read 200 thick film fields of Slide #1 and render a smear interpretation (Positive or Negative for asexual *Plasmodia* forms; gametocytes alone will NOT cause a slide to be classified positive). Results will be recorded on the appropriate DRF.

6. At each laboratory, Microscopists A and B will each identify parasites by species, and quantify parasites will using thick smears. Parasite density will be calculated by counting the number of asexual parasites (not gametocytes) per 200 WBCs and multiplying by WBCs per mcl. WBC count will be obtained by using the QBC® AutoReadPlus or COULTER AC-T10, or manual count as a back up.

7. In the event of discordant slide interpretations between Microscopists A and B, a third expert microscopist, Microscopist C, will examine both Slide #1 and Slide #2. Microscopist C will provide the final interpretation for the blood smears and will record results on the DRF.

8. Concordance / Discordance

Disconcordant blood smear interpretation results are disagreements between Microscopists A and B as to presence of parasitemia, quantitation of asexual parasitemia, or *Plasmodia* species identification.

Concordance is defined as:

Agreement about presence of asexual forms of *Plasmodia* on smear
-Agreement about species of *Plasmodia* present on smear
-Agreement on level of parasitemia within a factor of 2. For parasitemias of less than 500 per mcl, agreement within a factor of 3 is satisfactory. For parasitemias greater then 50,000 per mcl, an absolute difference of no more than 20,000 parasites per mcl will be tolerated.

Discordance is defined as absence of concordance on any one of the above three points

9. At least 5% of blood smears for which Microscopists A & B have given concordant readings will also be read by Microscopist C for quality control purposes. For this purpose, Microscopist C will examine Slide 1 only. In event of discordance between the quality control reading of Microscopist C and the concordant interpretations of Microscopists A and B, Microscopist A & B's interpretation will be used as the final microscopy reading. Microscopist C will record results on the DRF, and should annotate the comments section on the DRF with "QC".

10. To determine the "final micro" result, we compare concordant A and B readings and report an average of the two quantifications. We expect a small percentage of field read smears will be discordant with the final microscopists A&B reads. The field readings will be performed by a different microscopist than the final "study" reads to ensure blinding.

Attachment 14: Malaria Smear Interpretation DRF. Microscopist A: slide 1
WRAIR Protocol No. 719, CQ/PS resistance in the Ampage 1 of 1 Patient ID No:

Date sample collected : __ __ / __ __ / 98 Date slide read: __ __ / __ __ / 98 WBCs/mcl: ________
D D M M D D M M

Is slide positive or negative for malaria parasites? Negative (code = 0)Positive (code = 1)

Slide Interpretation (First species): 1. Pf 2. Pv 3. Pm 4. Po 5. Indeterminate

No. Asexual Parasites Counted	No. Gametocytes Counted	No. WBCs Counted	Asexual Parasites per mcl	Gametocytes per mcl	Morphology Code
					1. Asexual forms only<br>2. Sexual forms only<br>3. Both asexual and sexual forms present

No. asexual par. ____ X WBCs / mcl ____ = ____ Asexual parasites/mcl No. Gametocytes ____ X WBCs / mcl ____ = ____ gametocytes / mcl
No. of WBCs ____ No. of WBCs ____

Comments:

Slide Interpretation (Second Species): 1. Pf 2. Pv 3. Pm 4. Po 5. Indeterminate

No. Asexual Parasites Counted	No. Gametocytes Counted	No. WBCs Counted	Asexual Parasites per mcl	Gametocytes per mcl	Morphology Code
					1. Asexual forms only<br>2. Sexual forms only<br>3. Both asexual and sexual forms present

No. asexual par. ____ X WBCs / mcl ____ = ____ Asexual parasites/mcl No. Gametocytes ____ X WBCs / mcl ____ = ____ gametocytes / mcl
No. of WBCs ____ No. of WBCs ____

Comments:

DRF Completed by: Initials: ________ Date: ________ Data entry #1: Initials: ________ Date: ________
Investigator review: Initials: ________ Date: ________ Data entry #2: Initials: ________ Date: ________

Final Smear Interpretation: 0 = Neg 1= positive for one species only 2=positive for > one species (mixed infection)
Final Morphology :

3= Pf asexual monoinfection 4=Pf gametocytes only 5=Pf asexual and sexual forms present 6= Pv asexual monoinfection

7= Pv gametocytes only 8=Pv asexual and sexual forms present 9=mixed Pf / Pv infection 10=other

Final Verification: Initials: Date:

1. Asexual parasites will be counted and reported as number of asexual parasites per mcl of whole blood. Gametocytes will be counted and reported separately in the appropriate boxes in data report forms (attachment 14). Counts of parasites, WBCs, and gametocytes will be tallied on manual counters by the microscopists. If the slide shows a mixed infection, then separate counts will be made for each species. All parasites seen in the representative fields should be counted. If the microscopist reaches the 200 WBC count and there are more WBCs in the field of view, those should counted.

A) If, after 200 white blood cells (WBCs) are counted, 10 or more asexual stage parasites have been identified, record results on the appropriate data report form, indicating the number of parasites per number of WBCs counted.

B) If, after 200 white blood cells (WBCs) are counted, 9 or less asexual stage parasites have been identified, continue counting until 500 WBCs have been counted and record the parasites per 500 WBCs. Record results on the appropriate data report form, indicating the number of asexual parasites per number of WBCs counted.

2. In either case, the parasite count in relation to the WBC count can be converted to parasites per mcl by the mathematical formula:

$$\frac{\text{Number of asexual parasites}}{\text{Number of WBCs}} \times \text{WBC count (WBCs/mcl)} = \text{Number of asexual parasites per mcl}$$

For example, if 506 asexual parasites are counted per 209 WBCs and the volunteer's WBC count is 5.1 WBCs per mm^3 (as measured in the QBC® hematology system or Coulter counter) then, the number of asexual parasites per mcl is:

$$\frac{\text{506 asexual parasites enumerated}}{\text{209 WBCs enumerated}} \times \text{5100 WBCs per mcl} = \text{12,347 asexual parasites pr mcl}$$

3. If an automated WBC count cannot be made using the QBC® hematology system or Coulter counter, a manual WBC count will be performed using a standard Neubauer counting chamber if logistically feasible.

4. If it is not possible to perform a WBC count then an arbitrary count of 8,000 WBCs per mcl will be used.

Version dated May 1998

Summary: This SOP is used for cryopreserving and thawing malaria parasites obtained from patients. There is more than one accepted method depending on investigator preferences.

A. DMSO Method:

Adapted from Pavanand, et al., 1974 (*J. Parasitol.* 60:537-539).

1. Materials:

1) Sterile Dulbeccos phosphate buffered saline (PBS)
2) Sterile cryopreservation tubes
3) Sterile dimethylsulfoxide (DMSO)
4) Sterile 15 ml conical centrifuge tubes
5) Centrifuge

2. Preparation of Cryopreservation Fluid:

1) Aseptically add 6.8 ml of Dulbeccos PBS and 1.2 ml of DMSO to a sterile 15 ml conical centrifuge tube.

2) Mix the solution thoroughly and store in a refrigerator (if not used immediately).

3. Method:

1) Aseptically pipette at least 2 ml of heparinized whole blood into a sterile 15 ml conical centrifuge tube.

2) Centrifuge at 500 x g for 7 minutes. If your centrifuge is not calibrated, spin at a speed high enough to create a red cell pellet.

3) Remove the supernatant and add the PBS-DMSO mixture dropwise. For example, to a 1 ml packed cell volume of red blood cells, add 4 ml of the cryopreservation fluid. See the attached chart to determine the volume of cryopreservation fluid to add for various packed cell volumes.

4) The addition of cryopreservation fluid to the red cell pellet is the most critical step of the procedure. It is very important to add the PBS-DMSO mixture slowly and to mix frequently. For example, after adding 2-3 drops of cryopreservation fluid, you should mix the contents gently by tapping the tube with your finger. Then add 2-3 additional drops and repeat the mixing. By slowly adding the cryopreservation fluid, you ensure the least amount of subsequent hemolysis when the contents are thawed.

5) Once all the cryopreservation fluid has been added, then aliquot the mixture into cryovials and immediately place into liquid nitrogen (vapor phase). Do not pipette less than 0.5 or more than 1.8 ml into a single cryovial. If possible, prepare two or more cryovials for each parasite isolation.

6) All cryovials should be labeled appropriately with patient identification (e.g., study and patient number) and date of collection. Keep all vials frozen in liquid nitrogen until ready for use.

Chart to Determine Volume of Cryopreservation Fluid to Add to Packed Red Blood Cells

Packed Cell Volume	Cryopreservation Fluid Volume to Add	Final Volume
0.1 ml	0.4 ml	0.5 ml
0.2 ml	0.8 ml	1.0 ml
0.3 ml	1.2 ml	1.5 ml
0.4 ml	1.6 ml	2.0 ml
0.5 ml	2.0 ml	2.5 ml
0.6 ml	2.4 ml	3.0 ml
0.7 ml	2.8 ml	3.5 ml
0.8 ml	3.2 ml	4.0 ml
0.9 ml	3.6 ml	4.5 ml
1.0 ml	4.0 ml	5.0 ml

B. ACD Method of Cryopreservation:

1. Collect blood using acid citrate dextrose (ACD) solution 1:9
2. Centrifuge at 350 G for 5 minutes
3. Aspirate supernatant
4. Slowly add 1/3 volume of Glycerolyte 57 while swirling
5. Let stand for 5 minutes
6. Slowly add 4/3 volumes of Glycerolyte 57 with swirling
7. Aliquot into cryovials (ca. 0.1 - 0.5 ml)
8. Freeze at -80 degrees C overnight
9. Transfer to liquid nitrogen long term storage

EXAMPLE: volume of packed rbc's = 0.9 ml
1/3 volume = 0.3 ml
4/3 volume = 1.2 ml

C. Thawing of malaria-infected rbc's

1. Thaw quickly in 37 degree water bath without shaking
2. Add 0.1 ml of 12% NaCl slowly with gentle swirling
3. Let stand at room temp for 2 minutes
4. Add 10 ml of 1.6% NaCl slowly with swirling
5. Let stand at room temp for 5 minutes
6. Centrifuge at ca. 350 G for 5 minutes
7. Aspirate supernatant; add 10 ml 0.9% NaCl/ 0.2% dextrose slowly with swirling
8. Let stand at room temp for 5 minutes
9. Centrifuge at ca. 350 G for 5 minutes
10. Aspirate supernatant; add media/cells as desired

Attachment 17: Doses for Infants and Children of anti-malrial drugs according to body weight, expressed as fractions of tablets
WRAIR Protocol No. 719, CQ/PS resistance in the Amazon

WEIGHT (kg)	CHLOROQUINE			CHLOROQUINE			SULFONAMIDE/ PYRIMETHAMINE
	Tablets 100 mg *			Tablets 150 mg *			Tablet 500 mg S + 25 mg P
	Day 0	Day 1	Day 2	Day 0	Day 1	Day 2	Day 0
5	1 / 2	1 / 2	1 / 4	1 / 2	1 / 4	1 / 4	1/4
6	1 / 2	1 / 2	1 / 2	1 / 2	1 / 4	1 / 4	1/4
7	3 / 4	1 / 2	1 / 2	1 / 2	1 / 2	1 / 4	1/2
8	3 / 4	3 / 4	1 / 2	1 / 2	/ 1 2	1 / 2	1/2
9	1	3 / 4	1 / 2	1 / 2	1 / 2	1 / 2	1/2
10	1	1	1 / 2	3 / 4	1 / 2	1 / 2	1/2
11	1	1	3 / 4	3 / 4	3 / 4	1 / 2	1/2
12	1	1	1	3 / 4	3 / 4	1 / 2	3/4
13	1 1/4	1	1	1	3 / 4	1 / 2	3/4
14	1 1/4	1 1/4	1	1	1	1 / 2	3/4
15	1 1/2	1 1/4	1	1	1	1 / 2	3/4
16	1 1/2	1 1/2	1	1	1	3 / 4	3/4
17	1 3/4	1 1/2	1	1	1	1	3/4
18	1 3/4	1 3/4	1	1	1	1	1
19	2	1 3/4	1	1 1/4	1	1	1
20	2	2	1	1 1/4	1 1/4	1	1
21	2	2	1 1/4	1 1/4	1 1/4	1	1
22	2 1/4	2 1/4	1	1 1/2	1 1/2	3 / 4	1
23	2 1/2	2	1 1/4	1 1/2	1 1/2	1	1 1/4
24	2 1/2	2 1/2	1	1 1/2	1 1/2	1	1 1/4
25	2 1/2	2 1/2	1 1/4	1 1/2	1 1/2	1 1/4	1 1/4

* expressed as base

Attachment 18: Tables for calculating the minimal sample size according to the two stage lot quality assurance method with confidence level of 95% and power of 80%.

Po = 0.10

Pa=	n1+n2	d2	n1	d1
0.020	59	2	49	0
0.030	83	3	49	0
0.040	121	6	49	0
0.050	184	11	49	0
0.060	301	21	75	1
0.070	558	44	140	6
0.080	1303	112	326	20
0.090	5395	503	1349	109

Po = 0.15

Pa =	n1+n2	d2	n1	d1
0.030	38	2	31	0
0.045	53	3	31	0
0.060	77	6	31	0
0.075	117	11	31	0
0.090	191	20	48	1
0.105	354	42	89	5
0.120	824	106	206	19
0.135	3404	476	851	103

Po = 0.20

Pa =	n1+n2	d2	n1	d1
0.040	27	1	22	0
0.060	38	3	22	0
0.080	55	6	22	0
0.100	83	10	22	0
0.120	136	19	34	1
0.140	251	39	63	5
0.160	585	101	146	18
0.180	2409	449	602	98

Po = 0.25

Pa =	n1+n2	d2	n1	d1
0.050	21	1	16	0
0.075	29	3	16	0
0.100	42	5	16	0
0.125	63	10	16	0
0.150	103	18	26	1
0.175	190	37	48	5
0.200	441	95	110	17
0.225	1812	422	453	92

Po =0.3

Pa =	n1+n2	d2	n1	d1
0.060	16	1	13	0
0.090	23	3	13	0
0.120	33	5	13	0
0.150	50	9	13	0
0.180	81	17	20	1
0.210	149	35	37	5
0.240	345	89	86	16
0.270	1413	395	353	86

Po = 0.35

Pa =	n1+n2	d2	n1	d1
0.070	13	1	10	0
0.105	19	3	10	0
0.140	27	5	10	0
0.175	40	9	10	0
0.210	65	16	16	1
0.245	120	33	30	4
0.280	276	83	69	15
0.315	1129	368	282	80

Po = 0.40

Pa =	n1+n2	d2	n1	d1
0.080	11	1	8	0
0.120	15	2	8	0
0.160	22	5	8	0
0.200	33	8	8	0
0.240	54	15	14	1
0.280	98	31	25	4
0.320	225	77	56	14
0.360	916	342	229	74

Po = 0.45

Pa =	n1+n2	d2	n1	d1
0.090	9	1	7	0
0.135	13	2	7	0
0.180	18	4	7	0
0.225	28	8	7	0
0.270	44	14	11	1
0.315	81	29	20	4
0.360	185	72	46	13
0.405	750	315	188	69
